# Genome-wide association study using whole-genome sequencing identifies risk loci for Parkinson’s disease in Chinese population

**DOI:** 10.1038/s41531-023-00456-6

**Published:** 2023-02-09

**Authors:** Hongxu Pan, Zhenhua Liu, Jinghong Ma, Yuanyuan Li, Yuwen Zhao, Xiaoxia Zhou, Yaqin Xiang, Yige Wang, Xun Zhou, Runcheng He, Yali Xie, Qiao Zhou, Kai Yuan, Qian Xu, Qiying Sun, Junling Wang, Xinxiang Yan, Hainan Zhang, Chunyu Wang, Lifang Lei, Weiguo Liu, Xuejing Wang, Xuebing Ding, Tao Wang, Zheng Xue, Zhentao Zhang, Ling Chen, Qing Wang, Yonghong Liu, Jiayu Tang, Xuewei Zhang, Shifang Peng, Chaodong Wang, Jianqing Ding, Chunfeng Liu, Lijuan Wang, Haibo Chen, Lu Shen, Hong Jiang, Xinyin Wu, Hongzhuan Tan, Dan Luo, Shuiyuan Xiao, Xiang Chen, Jieqiong Tan, Zhengmao Hu, Chao Chen, Kun Xia, Zhuohua Zhang, Jia Nee Foo, Cornelis Blauwendraat, Mike A. Nalls, Andrew B. Singleton, Jun Liu, Piu Chan, Houfeng Zheng, Jinchen Li, Jifeng Guo, Jian Yang, Beisha Tang, the Parkinson’s Disease & Movement Disorders Multicenter Database and Collaborative Network in China (PD-MDCNC), Zhenhua Liu, Hong Jiang, Piu Chan, Jinchen Li, Jifeng Guo, Beisha Tang

**Affiliations:** 1 Department of Neurology, Xiangya Hospital, Central South University, 410008 Changsha, Hunan China; 2 National Clinical Research Center for Geriatric Disorders, Xiangya Hospital, Central South University, 410008 Changsha, Hunan China; 3 Department of Neurology, National Clinical Research Center for Geriatric Diseases, Xuanwu Hospital of Capital Medical University, 100053 Beijing, China; 4 Department of Neurology, Ruijin Hospital, Shanghai Jiao Tong University School of Medicine, 200025 Shanghai, China; 5 Hunan Key Laboratory of Molecular Precision Medicine, Department of Oncology, Xiangya Hospital, Central South University, 410008 Changsha, Hunan China; 6 Department of Geriatrics, Xiangya Hospital, Central South University, 410008 Changsha, Hunan China; 7 Department of Neurology, The Second Xiangya Hospital, Central South University, 410011 Changsha, Hunan China; 8 Department of Neurology, The Third Xiangya Hospital, Central South University, 410013 Changsha, Hunan China; 9 Department of Neurology, Affiliated Brain Hospital of Nanjing Medical University, 210029 Nanjing, Jiangsu China; 10 Department of Neurology, The First Affiliated Hospital of Zhengzhou University, 450047 Zhengzhou, Henan China; 11 Department of Neurology, Union Hospital, Tongji Medical College, Huazhong University of Science and Technology, 430022 Wuhan, Hubei China; 12 Department of Neurology, Tongji Hospital, Tongji Medical College, Huazhong University of Science and Technology, 430030 Wuhan, Hubei China; 13 Department of Neurology, Renmin Hospital of Wuhan University, 430060 Wuhan, Hubei China; 14 Department of Neurology, First Affiliated Hospital, Sun Yat-sen University, 510080 Guangzhou, Guangdong China; 15 Department of Neurology, Zhujiang Hospital of Southern Medical University, 510280 Guangzhou, Guangdong China; 16 Health Management Center, Hunan Provincial Brain Hospital, 410021 Changsha, Hunan China; 17 Department of Neurology, Hunan Provincial Brain Hospital, 410021 Changsha, Hunan China; 18 Department of Health Management Center, Xiangya Hospital, Central South University, 410008 Changsha, Hunan China; 19 Institute of Aging & Tissue Regeneration, Renji Hospital, Shanghai Jiao Tong University School of Medicine, 200025 Shanghai, China; 20 Department of Neurology and Clinical Research Center of Neurological Disease, The Second Affiliated Hospital of Soochow University, 215004 Suzhou, Jiangsu China; 21 Department of Neurology, Guangdong Provincial People’s Hospital, Guangdong Academy of Medical Sciences, 510080 Guangzhou, Guangdong China; 22 Department of Neurology, National Center of Gerontology, Beijing Hospital, 100005 Beijing, China; 23 Key Laboratory of Hunan Province in Neurodegenerative Disorders, Central South University, 410008 Changsha, Hunan China; 24 Department of Epidemiology and Health Statistics, Xiangya School of Public Health, Central South University, 410028 Changsha, Hunan China; 25 Department of Social Medicine and Health Management, Xiangya School of Public Health, Central South University, 410028 Changsha, Hunan China; 26 The Department of Dermatology, Xiangya Hospital, Central South University, 410008 Changsha, Hunan China; 27 Centre for Medical Genetics & Hunan Key Laboratory of Medical Genetics, School of Life Sciences, Central South University, 410012 Changsha, Hunan China; 28 Lee Kong Chian School of Medicine, Nanyang Technological University Singapore, Singapore, 308232 Singapore; 29 Molecular Genetics Section, Laboratory of Neurogenetics, National Institute on Aging, National Institutes of Health, Bethesda, MD 20892 USA; 30 Center for Alzheimer’s and Related Dementias, National Institute on Aging, National Institutes of Health, Bethesda, MD 20892 USA; 31 Data Tecnica International, Washington, DC 20037 USA; 32 Diseases & Population (DaP) Geninfo Lab, School of Life Sciences, Westlake University, 310024 Hangzhou, Zhejiang China; 33 School of Life Sciences, Westlake University, 310024 Hangzhou, Zhejiang China; 34 National Clinical Research Centre for Geriatric Disorders, Department of Geriatrics, Xiangya Hospital, Central South University, Changsha, China

**Keywords:** Parkinson's disease, Diagnostic markers

## Abstract

Genome-wide association studies (GWASs) have identified numerous susceptibility loci for Parkinson’s disease (PD), but its genetic architecture remains underexplored in populations of non-European ancestry. To identify genetic variants associated with PD in the Chinese population, we performed a GWAS using whole-genome sequencing (WGS) in 1,972 cases and 2,478 controls, and a replication study in a total of 8209 cases and 9454 controls. We identified one new risk variant rs61204179 (*P*_combined_ = 1.47 × 10^−9^) with low allele frequency, four previously reported risk variants (*NUCKS1/RAB29*-rs11557080, *SNCA*-rs356182, *FYN*-rs997368, and *VPS13C*-rs2251086), as well as three risk variants in *LRRK2* coding region (A419V, R1628P, and G2385R) with genome-wide significance (*P* < 5 × 10^−8^) for PD in Chinese population. Moreover, of the reported genome-wide significant risk variants found mostly in European ancestry populations, the correlation coefficient (*r*_b_) of effect size accounting for sampling errors was 0.91 between datasets and 63.6% attained *P* < 0.05 in Chinese population. Accordingly, we estimated a heritability of 0.14–0.18 for PD, and a moderate genetic correlation between European ancestry and Chinese populations (*r*_g_ = 0.47, se = 0.21). Polygenic risk score (PRS) analysis revealed that individuals with PRS values in the highest quartile had a 3.9-fold higher risk of developing PD than the lowest quartile. In conclusion, the present GWAS identified PD-associated variants in Chinese population, as well as genetic factors shared among distant populations. Our findings shed light on the genetic homogeneity and heterogeneity of PD in different ethnic groups and suggested WGS might continue to improve our understanding of the genetic architecture of PD.

## Introduction

Parkinson’s disease (PD) is a common age-related neurodegenerative disorder, characterised by bradykinesia, postural instability, rigidity, and resting tremors. The clinical picture includes non-motor symptoms such as cognitive impairment, autonomic dysfunction, disorders of sleep, depression and hyposmia^1^. The pathological hallmarks of PD are loss of dopaminergic neurons and Lewy bodies in the substantia nigra^2^. In China, the number of individuals with PD is estimated to reach 4.94 million by 2030, accounting for half of global cases^3^. The exact causes of PD are still unclear, but genetic factors, environmental factors and aging play essential roles in pathogenesis of PD^4^.

Although pathogenic mutations in disease-causing genes play essential roles in PD, few patients carry pathogenic mutations^5,6^. Common variants with small effect size can be detected via genome-wide association studies (GWASs), and have been shown to affect a large proportion of genetic predisposition of PD^7^. Previous GWASs of PD in European ancestry, East Asian, and Latino populations comprising more than one million samples have identified over 80 risk loci with more than 90 independent risk variants, including in *SNCA*, *LRRK2*, *MAPT*, *BST1*, *GCH1*, *VPS13C*, and *TMEM175*^8–10^. Genetic heterogeneity among populations has been inferred from differences in risk loci, such as the overall genetic architecture defined by allele frequency and linkage disequilibrium (LD) patterns, and allelic heterogeneity between European ancestry and East Asian individuals^8,11^. GWAS data have pointed to several biological pathways involved in PD pathogenesis, such as mitochondrial function, lysosomal function, and endocytosis^8,11^. However, a large proportion of target subjects were of European ancestry, extrapolating these results to other populations such as Chinese becomes difficult and there is no large GWAS study of PD in Chinese population yet.

The present study explored the genetic architecture of PD with the aim of identifying risk loci responsible for its pathogenesis in the Chinese population. We conducted a GWAS in a total of 1972 PD cases and 2478 controls using whole-genome sequencing (WGS), as well as a replication study in 8209 cases and 9454 controls using multiplex PCR sequencing. We also compared genome-wide significant variants for PD, reported mostly in population of European ancestry, with our results of Chinese population. Finally, we estimated the heritability of PD, assessed its genetic correlation with other traits, and established a predictive model to evaluate at-risk individuals in the Chinese population.

## Results

### Cohort description and quality control

A total of 22,366 subjects were included in this study: 1980 patients with PD and 2516 matched controls in the GWAS cohort, plus 8294 cases and 9576 matched controls in the replication cohort. Details about genotype quality of GWAS and replication cohorts are provided in Supplementary Table 1 and Supplementary Table 2. The final subjects included in the statistical analysis after quality control (Supplementary Table 3) encompassed 1972 cases (the mean age at recruitment, 66.76 ± 7.08 years; the mean age at onset, 61.88 ± 6.93 years) and 2478 controls (the mean age at recruitment, 62.32 ± 7.11 years) in the GWAS cohort, and 8209 cases (the mean age at recruitment, 60.23 ± 11.20 years; the mean age at onset, 57.77 ± 11.95 years) and 9454 controls (the mean age at recruitment, 64.29 ± 9.68 years) in the replication cohort. Detailed demographic information is listed in Supplementary Table 4.

**Fig. 1:**
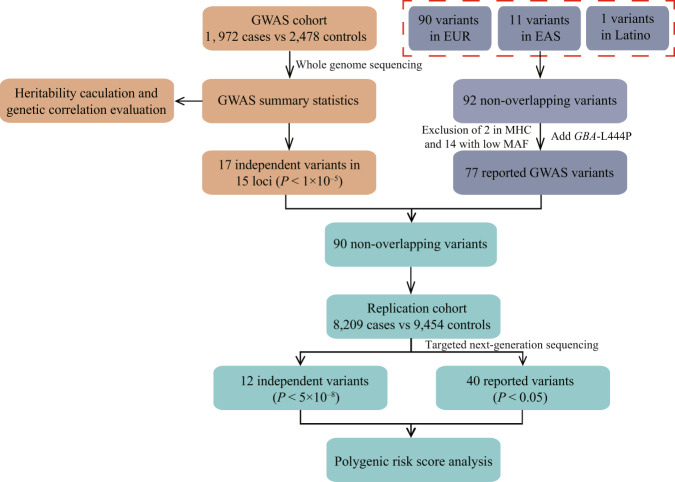
Workflow of the study. EUR European, EAS East Asian, MHC histocompatibility complex region, MAF minor allele frequency.

To explore the quality of WGS with the medium degree of depth, 54 samples from the GWAS cohort were randomly picked using Excel and subjected to WGS with a high degree of depth setting, whereas another randomly picked 39 samples were re-sequenced with the same WGS as GWAS cohort. Sequencing results were compared within sample pairs, revealing good genotype concordance between the analysed GWAS variants (Supplementary Fig. 1). The workflow of this study was showed in Fig. 1.

### Genome-wide association analysis

**Fig. 2:**
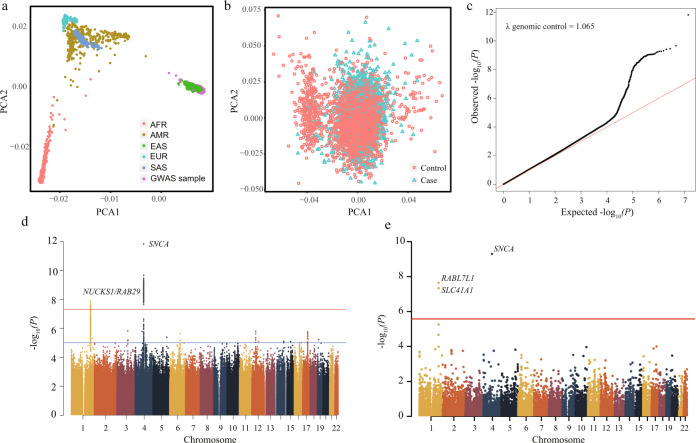
Association results of GWAS cohort. **a** PCA plot of samples projecting to the 1000 Genomes subjects. **b** PCA of our 1972 PD cases and 2478 controls in GWAS cohort. **c** The quantile–quantile (Q–Q) plot of the association results from the GWAS cohort. **d** Manhattan plot of *P* values on the −log_10_ scale for 6,910,086 SNPs in the GWAS cohort. **e** Manhattan plot of *P* values on the −log_10_ scale for genome-wide gene-based analysis in the GWAS stage.

**Fig. 3:**
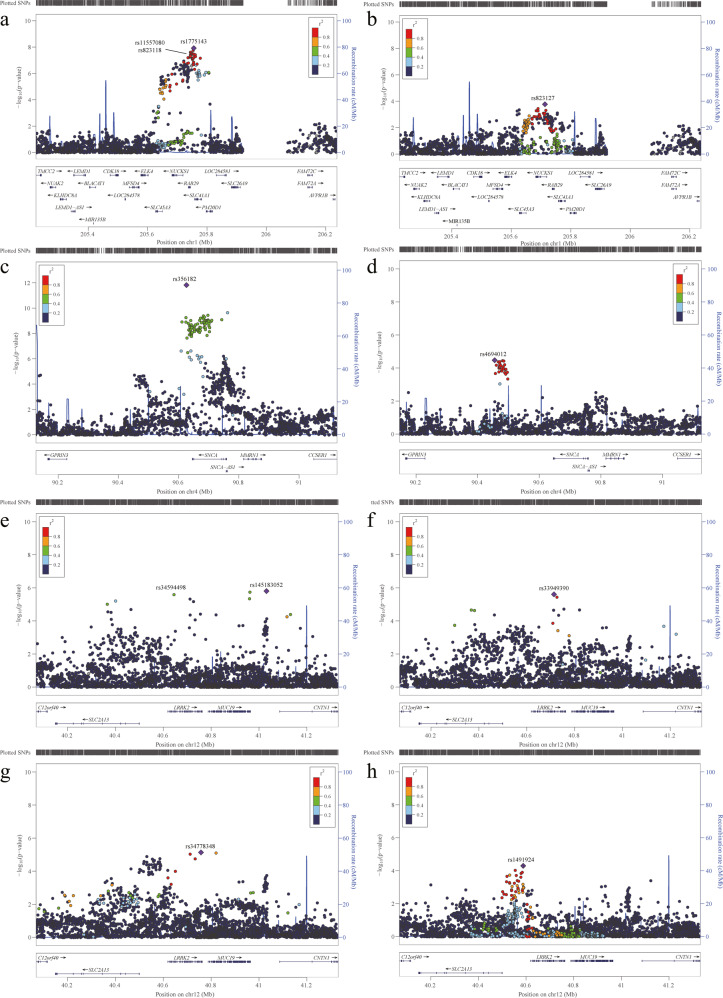
Regional plot of conditional analysis of reported loci with multiple independent signals. **a**, **b** Conditional analysis in *NUCKS1/RAB29* region. **c**, **d** Conditional analysis in *SNCA* region. **e**–**h** Conditional analysis in *LRRK2* region. Reference data of Asian population from 1000 Genomes were used for linkage disequilibrium calculation.

In the GWAS cohort, 6,910,086 autosomal single nucleotide polymorphisms (SNPs) passed the quality control threshold and were included in further analyses. Principal component analysis confirmed a good match between patients with PD and controls (Fig. 2a, b). A quantile-quantile plot indicated that population stratification had negligible effects on the statistical results (λ genomic control = 1.065, LD score intercept = 1.036; (Fig. 2c). Two known risk loci (*NUCKS1*/*RAB29* and *SNCA*) exhibited genome-wide significance (*P* < 5 × 10^−8^), and 13 risk loci exhibited suggestive significance (*P* < 1 × 10^−5^) in the GWAS analysis. Of the 13 suggestive risk loci, three were known (*LRRK2*, *FYN*, and *VPS13C*) and 10 were possibly novel (Fig. 2d; Supplementary Table 5). *NUCKS1/RAB29*, *SNCA*, and *LRRK2* regions were previously reported to harbour multiple independent signals, and conditional analysis of our GWAS data revealed probable multiple signals in *LRRK2*, but not in *NUCKS1/RAB29* and *SNCA* regions (Supplementary Table 6). In the *LRRK2* locus, three previously reported risk variants, rs34594498 (*LRRK2*-A419V), rs33949390 (*LRRK2*-R1628P), and rs34778348 (*LRRK2*-G2385R), were found to be independently associated with PD with suggestive significance (*P* < 1×10^−5^) (Fig. 3).

In the replication cohort, 17 independent risk variants in 15 risk loci were genotyped and analysed. In the combined analysis, eight of these SNPs showed associations exceeding the genome-wide significance threshold (*P* < 5.0 × 10^–8^; Table 1), including four reported GWAS SNPs, three risk variants in the *LRRK2* gene and one newly identified risk variant rs61204179.

**Table 1:** Association results of eight genome-wide significant variants in combined analysis.

Tag SNP	Position (hg19)	Nearest gene	Effect allele	Other allele	MAF_EAS	MAF_EUR	GWAS cohort			Replication cohort			Combined			
							Beta	se	*P*	Beta	Se	*P*	beta	Se	*P*	*P*_het_
rs11557080	1:205737739	*NUCKS1/RAB29*	G	A	0.457	0.855	−0.249	0.046	6.08E−08	−0.206	0.024	1.80E−18	−0.215	0.021	1.04E−24	0.399
rs356182	4:90626111	*SNCA*	A	G	0.332	0.650	−0.344	0.049	1.52E−12	−0.342	0.025	2.56E−42	−0.343	0.022	3.09E−53	0.970
rs997368	6:112243291	*FYN*	G	A	0.341	0.128	−0.160	0.046	5.37E−04	−0.109	0.023	3.39E−06	−0.119	0.021	1.11E−08	0.326
rs34594498	12:40646786	*LRRK2*	T	C	0.007	0	0.984	0.209	2.47E−06	0.855	0.108	2.64E−15	0.883	0.096	4.05E−20	0.586
rs33949390	12:40713845	*LRRK2*	A	G	0.028	0.0006	0.661	0.144	4.57E−06	0.503	0.076	4.02E−11	0.544	0.067	6.68E−16	0.343
rs34778348	12:40757328	*LRRK2*	C	G	0.029	0	0.452	0.110	3.71E−05	0.643	0.061	4.26E−26	0.608	0.053	3.22E−30	0.233
rs2251086	15:61997385	*VPS13C*	T	C	0.162	0.149	−0.191	0.061	1.77E−03	−0.158	0.030	1.56E−07	−0.164	0.027	1.19E−09	0.622
rs61204179	17:58139282	*HEATR6*	T	C	0.043	0	0.477	0.105	5.86E−06	0.242	0.054	7.91E−06	0.291	0.048	1.47E−09	0.047

MAF data were extracted from gnomAD database.

*Beta* regression coefficient, *se* standard error of beta, *P*_het_ the *P*-value of heterogeneity, *MAF* minor allele frequency, *EUR* European, *EAS* East Asian.

**Fig. 4:**
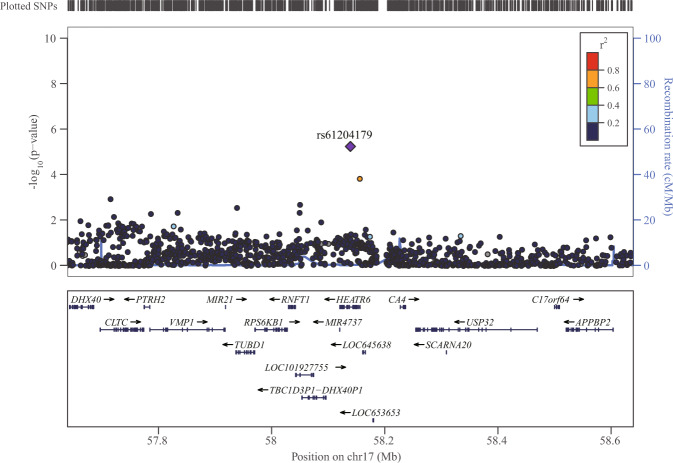
Regional plot of the rs61204179. Regional plot of the association signal of rs61204179 by LocusZoom. Reference data of Asian population from 1000 Genomes were used for linkage disequilibrium calculation.

The new risk variant (rs61204179) was located in the ninth intron of the *HEATR6* gene. This variant had a minor allele frequency of 4% in our population and low LD with other variants in the *HEATR6* region (*r*^2^ < 0.2 with most variants in the East Asian individuals of 1000 Genomes Project data, Fig. 4, Supplementary Fig. 2). Notably, rs61204179 was rare in the populations of European ancestry, making it a Chinese-specific PD risk variant, similar to the associated variants (A419V, R1628P and G2385R) in *LRRK2* gene. *HEATR6* shows high expression in multiple brain regions including midbrain (Supplementary Fig. 3) and was reported to be downregulated in the peripheral blood of patients with PD^12^.

### Gene-based association analysis

Besides the GWAS of common SNPs, we conducted genome-wide gene association analysis using GWAS data by MAGMA. MAGMA relies on converging evidence from multiple genetic variants in the same gene and can yield novel signals at a gene-based level. Here, it identified three associated genes (*RAB7L1*, *SLC41A1*, and *SNCA*), all of which were located in genome-wide significant loci by the GWAS (Fig. 2e; Supplementary Table 7).

### Validation of reported GWAS variants

**Fig. 5:**
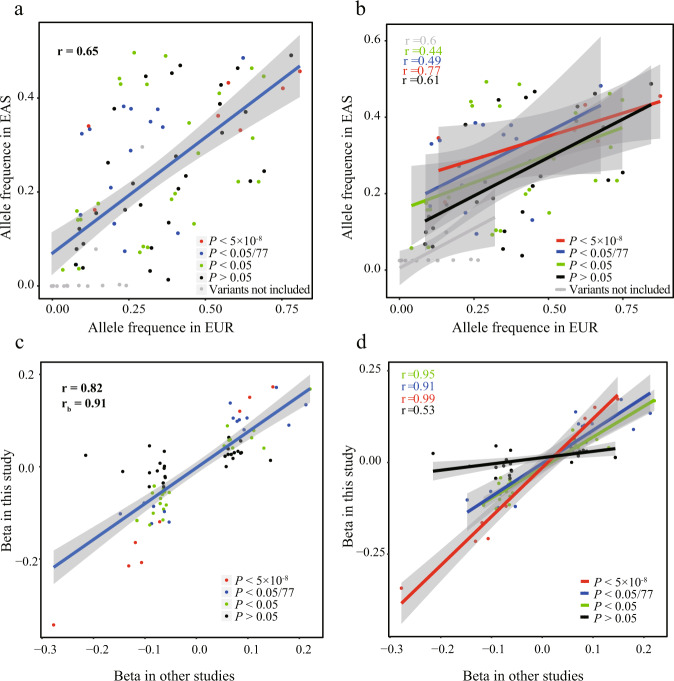
Correlation plots. **a**, **b** Allele frequency—Allele frequency plot showing the correlation of minor allele frequencies for reported genome-wide significant variants between European (*x*-axis) and East Asian populations (*y*-axis); allele frequencies were extracted from gnomAD database. **c**, **d** Beta-beta plot showing the correlation between effect sizes derived from the GWAS in which they were reported (*x*-axis) and effect sizes calculated in this study (*y*-axis); beta in other studies were from European population (Nalls, 2019) and East Asian population (Foo, 2020). Red dots denote variants with *P* < 5 × 10^−8^, blue dots denote variants with *P* < 0.05/77, green dots denote variants with *P* < 0.05, black dots denote variants with *P* > 0.05, and grey dots denote variants not included in replication cohort of this study. r shows the Pearson correlation coefficient and *r*_b_ shows the correlation coefficient correcting for errors in the estimated effects. EUR European, EAS East Asian.

To date, 78 loci and 90 independent variants have been reported in populations of European ancestry, 11 loci (11 variants) in the East Asian population, and one locus (one variant) in the Latino population^8–10^. Among the 92 independent PD-associated variants in the 80 non-overlapping loci, a high correlation of minor allele frequency was observed between the East Asian and European ancestry populations (*r* = 0.65, Fig. 5a, b). Fourteen variants with minor allele frequency < 0.5% in the East Asian population and two variants located in the major histocompatibility complex region, were excluded from the validation analysis. Besides, the *GBA*-L444P variant was added in validation given the reported genome-wide significant risk variant in *GBA* gene (N370S) in European ancestry populations was very rare in the Chinese population but *GBA*-L444P was reported to be associated with PD in Chinese.

Of the 77 reported variants included in our validation analysis, nine attained genome-wide significance in the combined data, 15 were significant after Bonferroni correction (*P* < 0.05/77), and 25 had *P* < 0.05 (a total of 63.6% variants were validated, Fisher’s exact test *P* < 2.84 × 10^−15^). All of the validated variants had the same effect direction as the reported, and of the 28 non-validated variants, 24 exhibited the same effect direction as reported previously (Supplementary Table 8), suggesting that more samples were needed to further validate these variants (sign concordance test *P* < 1.99 × 10^−9^). Specifically, rs11557080 and rs823118 were reported to be independent risk variants in the *NUCKS1/RAB29* locus in populations of European ancestry, but they appeared in high LD in our data (*r*^2^ = 0.94), and conditional analysis showed that only one signal remained significant (Supplementary Table 8). Moreover, the estimation of variants effect correlation (r) between our data and existing data^8,9^ was 0.82, and the correlation estimate (*r*_b_) was 0.91 when accounting for errors in the variant’s effects (Fig. 5c, d), suggesting a large proportion of GWAS markers discovered in Europeans were likely replicable in the Chinese population. Nevertheless, some differences between the associated sites still persist.

### Heritability, genetic correlation and MR

Using the genetic data of the GWAS cohort, the estimated heritability of PD in the Chinese population was 0.140 (se = 0.047) by LDSC and 0.184 (se = 0.041) by GCTA-GREML, assuming a global prevalence of 0.5%. These values seemed lower than the estimate of 0.22 for populations of European ancestry^8^, but the heritability remained comparable between two populations given the relatively large confidence interval of heritability estimates.

Using our GWAS data and publicly available PD-GWAS summary statistics, we found that the European ancestry and Chinese populations showed an intermediate genetic correlation (*r*_g_ = 0.47, se = 0.21), suggesting a certain amount of population heterogeneity in PD. This result was in line with the validation outcomes of GWAS variants and further confirmed the need for genome research on PD among different populations.

We further analysed possible genetic correlations between Chinese patients with PD and other phenotypes in East Asian populations. Using publicly available GWAS summary data originating mainly from the BioBank Japan Project, 157 traits were analysed, but no significant genetic correlation was found after Bonferroni correction. However, height, type 2 diabetes, rheumatoid arthritis, systemic lupus erythematosus, and gamma-glutamyl transferase showed suggestive association with PD (*P* < 0.05) (Supplementary Table 9).

Additional MR analysis between PD and phenotypes with suggestive genetic correlation refuted any significant causal relationship (*P* > 0.05) (Supplementary Table 10).

### Polygenic risk score analysis

**Fig. 6:**
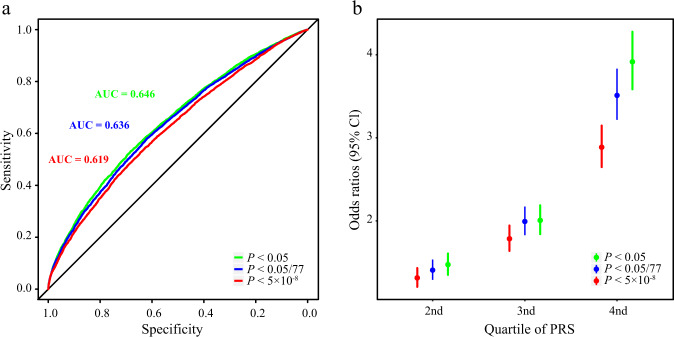
Predictive model. **a** Polygenic risk score (PRS) receiver-operator curves for variants with different significance levels. **b** Odds ratio of developing PD for each quartile of the PRS compared with the lowest quartile of genetic risk. Red lines and dots correspond to data calculated from variants with *P* < 5 × 10^−8^, blue lines and dots correspond to data calculated from variants with *P* < 0.05/77, and green lines and dots correspond to data calculated from variants with *P* < 0.05.

Using the GWAS cohort as a training dataset for effect size calculation and the validation cohort as test data, we calculated polygenic risk scores based on three sets of SNPs stratified by significance level. For 12 genome-wide significant variants, the proportion of explained genetic liability of PD was 16% based on our current heritability estimates. Moreover, 12 genome-wide significant variants (*P* < 5 × 10^–8^) and 15 variants with (*P* < 0.05/77) explained 20% of PD heritability, while 52 variants with *P* < 0.05 accounted for 23% of heritability (Fig. 6a). In the weighted polygenic risk score distribution based on different single nucleotide variant sets, we observed a 2.8-fold and 3.9-fold difference in risk between the top and bottom 25% of the test population (Fig. 6b).

## Discussion

We conducted a GWAS and a replication study in a large Chinese cohort, identified a new PD-related locus, and confirmed multiple independent risk variants with genome-wide significance in the *LRRK2* locus. In addition, a large proportion of reported GWAS variants were validated in our data. We constructed polygenic risk models to better predict the risk of PD and also systematically assessed the genetic correlation of PD in different populations, and in relation to other phenotypes among East Asians. Overall, these analyses may provide new insights on the impact of genetic variants on the pathogenesis of PD.

SNP arrays and WGS have been shown similarly efficient at surveying common variants in the genome^13^. Here, we employed the WGS method for genotyping our GWAS cohort, because it captured significant signals with low minor allele frequency, which were not well imputed in commonly used SNP arrays^8,9^. This was the case of the newly identified risk locus in the *HEATR6* gene and multiple independent risk variants located in the *LRRK2* region, supporting the potential use of WGS for future GWASs. On the other hand, the newly identified locus in the *HEATR6* gene could not be validated in the published PD GWAS datasets of Asian populations^9,14,15^ due to the methodology differences. In addition, the tag SNP of the new risk locus was a rare variant in populations of European ancestry and, therefore, it could not be validated in PD GWAS datasets of European ancestry populations either^8,15^. Given that most currently used functional annotation databases are derived from populations of European ancestry^16–18^, while the corresponding databases for East Asian populations are still too narrow in scope^19^, it is hard to comprehensively explore the possible regulatory mechanism of this locus. Based on data from European ancestry populations^20–22^, there is few genomic regulatory elements in the region harbouring this locus. *HEATR6* encodes the HEAT repeat containing protein 6, which have 37–47 amino acid motifs called HEAT repeats forming repetitive arrays of short amphiphilic α-helices. While the functions of the HEATR6 remains unclear, HEAT repeat proteins mediate important protein-protein interactions involved in cytoplasmic and nuclear transport, microtubule dynamics and chromosome segregation^23^. Several HEAT repeat proteins were reported to cause neurological diseases^23–25^. *HEATR6* showed high expression in multiple brain regions including midbrain, moreover, Heatr6 were reported to be altered in neurological diseases^26,27^ and downregulated in the peripheral blood of patients with PD^12^. We speculate the new risk variant rs61204179 may mediate the risk of PD through HEATR6, but functional studies are required to further confirm the relationship between this locus and PD.

Multiple independent risk variants were identified by GWASs in the *LRRK2* region of European ancestry populations^8,28^, and this observation was confirmed in Chinese with *LRRK2*-A419V, *LRRK2*-G2385R, and *LRRK2*-R1628P showing associations independently. However, the risk variants of *LRRK2* exhibited significantly different incidence across populations. For example, the *LRRK2*-G2019S which was identified in European ancestry populations is very rare in the Chinese population; whereas multiple independent variants identified in the present study are rare in the European ancestry populations^7,8^. In addition, although the reported GWAS signal for rs76904798, located in the 5ʹ untranslated region of *LRRK2*, was common in both populations, no significant association was found in the Chinese population, indicating substantial population heterogeneity in the *LRRK2* locus. This finding is of relevance for future studies, which should further explore PD-related variants in the *LRRK2* region, such as finding more disease-associated variants and fine mapping of the causal variants across different populations.

We systematically validated the reported GWAS variants and found that those identified in populations of European ancestry were well validated in our population. In fact, the correlation of effect size between European ancestry and East Asian populations was significantly better than the previously published correlation (0.82 vs. 0.44)^9^. The correlation was even higher (0.91) after applying *r*_b_ method which estimate the correlation of estimated SNP effects accounting for estimation errors. Moreover, after the stratification of variant’s significance, the effects of validated variants were better correlated between datasets. This result pointed to the genetic homogeneity of PD between the two populations and indirectly confirmed the success of our methodology. One of the two risk variants identified in Eastern Asian have not been validated in our study (*SV2C*-rs246814, *P* = 0.49), despite the strong association of *SV2C*-rs246814 was observed in further replication study in both European ancestry population and pooling dataset of worldwide population^29^. Another risk variant was validated in our data (*WBSCR17*-rs9638616, *P* = 0.047), which was consistent with former studies^9,29^. Future studies are required to investigate PD among populations inhabiting different regions of East Asia.

No significant genetic correlation was found between traits describing the East Asian population and the PD in Chinese population, however, suggestively significant correlated traits found in this study (including type 2 diabetes, autoimmune disorders and height) may be informative for further epidemiological, clinical, and pathophysiological researches^30^. The PRS model was constructed in this study, but the overall performance of PD prediction was limited, and there was no significant improvement compared with the previous studies.

This study has some limitations. First, although the total sample size exceeded 20,000, this study had moderate power because the subjects were divided into GWAS and validation cohorts, resulting in limited power to detect variants with small or moderate effects. This may be the reason for the limited number of identified new loci and the high false-positive rate of nominally significant GWAS signals. Second, we only validated tag SNPs in the associated loci, but not in the entire locus, which could result in the loss of valuable information for fine-mapping based on different LD structures within populations. Third, the newly identified risk signal has not been verified by functional studies, and requires further validation in larger cohorts.

In conclusion, this GWAS using WGS identified PD risk loci in Chinese population and validated shared genetic factors among PD from different populations. These findings further emphasise both the genetic homogeneity and heterogeneity of PD in disparate populations, and suggested the potential of WGS in improving our understanding of the disease’s genetic architecture.

## Methods

### Participants

A total of 10,274 patients with PD and 12,092 control subjects free from neurological disorders were recruited from study groups participating in the Parkinson’s Disease and Movement Disorders Multicenter Database and Collaborative Network in China (http://pd-mdcnc.com)^31^ and centres across China. The GWAS included 1980 cases with PD and 2516 matched controls due to the costs of WGS and the numbers of study participants recruited during the GWAS phase. Another 8,294 cases and 9,576 controls were included in the replication study. Each patient was diagnosed by two neurologists specialised in movement disorders according to either the Movement Disorder Society Clinical Diagnostic Criteria^32^ or the United Kingdom Parkinson’s Disease Society Brain Bank Clinical Diagnostic Criteria^33^ in recruiting sites. Blood samples were collected, and genomic DNA was prepared according to standard procedures. The study was approved by the ethics committees of Xiangya Hospital of Central South University and other centres including Xuanwu Hospital of Capital Medical University, Ruijin Hospital of Shanghai Jiao Tong University School of Medicine, Affiliated Brain Hospital of Nanjing Medical University, Union Hospital of Huazhong University of Science and Technology, the First Affiliated Hospital of Zhengzhou University and First Affiliated Hospital of Sun Yat-sen University. Written informed consent was obtained from the participants or their legal guardians.

### Next-generation sequencing and variants calling

For the GWAS, WGS was performed on the Illumina NovaSeq 6000 sequencing platform with 150-bp pair-end reads, followed by preparation of an Illumina DNA library using genomic DNA sheared to 100–1000 bp. The mean WGS depth was 13× per individual in the GWAS cohort.

For the replication study, a single multiplex PCR was used to amplify the amplicons of candidate variants from our GWAS, as well as variants reported in previous GWASs on European ancestry, East Asian, and Latino populations^8–10^, followed by sequencing on an Illumina platform. The mean depth was 2000× per individual in the replication cohort.

Variant calling was conducted on all samples using BWA^34^ and GATK4^35^. Clean reads were aligned to the GRCh37 human reference genome (hg19) using the BWA mem tool to produce SAM files. The SAMtools^36^ view option was used convert SAM files into BAM files. MarkDuplicates was used to mark PCR duplicates with a REMOVE_DUPLICATES parameter setting of false. The BaseRecalibrator generated a recalibration table using known dbSNP and 1000 Genomes Project VCF resources. ApplyBQSR generated the recalibrated final BAM files for HaplotypeCaller. The gVCF file for each sample was obtained and combined into joint VCF files using GATK4 GenomicsDBImport and GenotypeGVCFs, following the suggested pipelines. We calculated the VQSLOD value of each single nucleotide variant (SNV) and insertion-deletion (INDEL) variant by setting the max-Gaussian value to 5. SNVs were annotated with ReadPosRankSum, MQRankSum, DP, QD, FS, and SOR; whereas INDELs were annotated with ReadPosRankSum, DP, QD, and FS. In the ApplyVQSR filtering step, the true sensitivity level for INDELs and SNVs was set to 99.0 and 99.6, respectively. All variants that passed the filter were retained for downstream analyses. We conducted LD-based genotype refinement for low-confidence genotypes and missing sites in WGS data using BEAGLE v5.1^37^ with default settings.

### Quality control

For individual and variant quality control of WGS data, the data were recoded into binary PLINK input format using PLINK v1.9^38^. Variant quality control was accomplished by keeping all autosomal SNPs with minor allele frequency ≥ 0.01 which may have sufficient power to detect the associations given the sample size in GWAS cohort^13^, while removing variants deviating from the Hardy-Weinberg equilibrium (*P* < 1 × 10^−4^). Individuals and variants that passed the quality control thresholds were subjected to further analyses. Individuals were excluded if they showed conflicting sex assignments between inferred sex and self-reported sex, deviating heterozygosity/genotype calls (±3 standard deviations), or cryptic relatedness (identity by descent > 0.15). The remaining samples were assessed for population outliers and stratification by principal component analysis using PLINK v1.9, and those that showed divergent ancestry were excluded. For the replication cohort, variants with low-quality genotypes (Phred-scaled genotype quality score 30), low call rates (missing rate > 5%), or departure from Hardy–Weinberg equilibrium (*P* < 1 × 10^−4^) were excluded. Samples with low genotype call rates (missing rate > 5%) were removed from further analysis. We also did principal component analysis using genotyped variants not associating with PD and excluded the outliers (Supplementary Fig. 4).

We performed WGS with a high degree of depth setting (30× depth) on 54 GWAS samples which were randomly picked using Excel from the entire list of control subjects to estimate the calling accuracy of WGS with medium depth (13× depth). Moreover, WGS with medium depth (13×) was performed again on 39 randomly picked GWAS control samples to estimate calling consistency. We computed calling concordance between matched samples for genotypes of all GWAS-analysed variants and non-reference genotypes of GWAS variants using previously specified formulae^39^.

### Association testing and combined analysis

For GWAS, logistic regression analysis was conducted in PLINK v1.9 to test the differences in allele dosage between patients with PD and controls. An additive genetic model adjusted for sex, age, and population substructure based on the first five principal components was employed. Conditional analyses were conducted by adding in-locus tag SNP, which was the most significant variant in our data or reported variant in linkage with the most significant variant, to logistic regression as covariates until no variants within the locus reached suggestive significance (*P* < 1 × 10^−5^). SNPs reached suggestive significance within the corresponding locus in the GWAS, and previously reported variants in GWASs were included for validation. In the replication study, logistic regression analyses in PLINK v1.9 were conducted on genotype dosages with sex, age and first two principal components as covariates.

To improve the statistical power of the validated variants, we used meta-analysis to combine the association results from the GWAS and replication study using METAL^40^ with an inverse variance-based model. The extent of heterogeneity was assessed using I^2^ and *P*-values of the Q statistics calculated in METAL^41^.

### Gene-based association analysis

SNP-based *P*-values from the GWAS were used as inputs for gene-based analysis of common variants. We used all 19,427 protein-coding genes from NCBI 37.3 (hg19) gene definitions as the basis for a genome-wide gene association analysis in MAGMA^42^. The MAGMA approach is based on a multiple linear principal component regression model. By projecting the multivariate LD matrix of SNPs in a gene estimated from the 1000 Genomes Phase 3 Asian reference population^43^, the principal components explaining genetic variation were extracted first. These principal components were used as predictors of PD under a linear regression framework. MAGMA then employed Fisher’s test to compute *P*-values for association testing. A stringent Bonferroni correction was applied to account for multiple testing.

### SNP-based heritability analysis

We used linkage disequilibrium score regression (LDSC)^44^ and univariate genome-wide complex trait analysis- genomic-relatedness-based restricted maximum-likelihood (GCTA-GREML)^45^ with default parameters and the same covariates as GWAS to estimate the heritability of PD. East Asian LD scores were downloaded from https://github.com/bulik/ldsc. The LDSC method derives the heritability of PD by regressing an SNP’s association statistic onto its LD score (the sum of squared correlations between the minor allele count of the SNP and the minor allele count of every other SNP). GCTA-GREML methods estimate heritability by calculating the proportion of variation in phenotypes explained by common SNPs using individual phenotypic and genotypic information.

### Genetic correlation and variant effect correlation

POPCORN^46^ was used to investigate the genetic correlation of PD between populations of European ancestry and East Asians. POPCORN uses a Bayesian approach, which assumes that genotypes are drawn separately from each population and that effect sizes follow the infinitesimal model. Genetic correlations in POPCORN were computed in the ‘genetic effect’ mode, which estimated the correlation based on LD covariance scores and effect sizes from summary statistics. We used bivariate LDSC to investigate the genetic correlations of PD with multiple traits and diseases in East Asian populations based on data from the BioBank Japan Project (http://jenger.riken.jp/en/result) and the GWAS catalogue^47^. Pearson correlation analysis and *r*_b_ method^48^ which correct sampling errors in the estimated SNP effects were used to estimate the correlation of SNP effects between reported GWASs and our data.

### Mendelian randomisation

Mendelian randomisation (MR) was used to assess the potential causal role of traits showing significant genetic correlations with PD. A two-sample MR approach was applied using genetic effect estimates for SNPs presenting genome-wide significance for PD, and those for the risk of associated traits. The analysis was performed using the MendelianRandomization package^49^ in R based on two different strategies. According to the inverse variance weighted (IVW) MR method, which assumed that all SNPs were valid instrumental variables, conventional linear regression analysis of Wald ratios for each SNP was undertaken and weighted by the estimated inverse variance. This method constrains the regression when βZX and βZY are equal to zero. According to the MR-Egger regression, horizontal pleiotropy was tested to provide a causal estimate in the presence of pleiotropy. As in the IVW method, βZY is plotted against βZX. However, the intercept is not fixed; therefore, the deviation from the origin provides evidence for pleiotropic effects in the corresponding direction. In the absence of unbalanced pleiotropy, if SNPs are viewed individually, βIV values are distributed symmetrically around the point estimate, as demonstrated by a funnel plot.

### Polygenic risk prediction

Predictive modelling was performed using the polygenic risk score in the replication samples. Weighted polygenic risk scores were calculated based on SNP significance in the combined analysis and their effect size in the GWAS. Areas under the receiver operating characteristic curve were calculated by comparing the observed case/control status and the polygenic risk score calculated using PRSice2^50^ profiling in a standard weighted allele-dose manner. Equations from Wray and colleagues^51^ were used to estimate the proportion of genetic liability accounted by SNP sets assuming a global prevalence of 0.5%.

### Reporting summary

Further information on research design is available in the Nature Research Reporting Summary linked to this article.

## Supplementary information

SUPPLEMENTAL MATERIAL

Reporting Summary

## Data Availability

The summary data of this GWAS can be accessed after an approved application to the Open Archive for Miscellaneous Data (OMIX) of National Genomic Data Center (NGDC). The accession code is OMIX002795.

## Code availability

The related codes and scripts for the study will be made available upon reasonable request to the corresponding author.
