# Supplementary material for: Genome-wide association study using whole-genome sequencing identifies risk loci for Parkinson’s disease in Chinese population
Source: NPJ Parkinsons Dis. 2023 Feb 9;9:22. doi: 10.1038/s41531-023-00456-6 (PMC9911746; doi:10.1038/s41531-023-00456-6)
Supplement: Supplementary file 1 — SUPPLEMENTAL MATERIAL [file 41531_2023_456_MOESM1_ESM.pdf]

Supplementary Table 1. Genotyping quality of whole genome sequencing of GWAS cohort

**Items (N = 4496)**	**Mean ± Standard deviation**
raw total sequences (Mega bases)	271.27 ± 61.01
reads properly paired (%)	96.48 ± 1.55
reads duplicated (%)	6.97 ± 2.72
average quality	34.51 ± 1.709
insert size average	394.72 ± 27.00
insert size standard deviation	98.32 ± 28.98
mean depth (×)	13.99 ± 3.10
coverage ⩾ 1 (%)	99.13 ± 0.35
coverage ⩾ 5 (%)	94.63 ± 2.21
coverage ⩾ 10 (%)	67.70 ± 12.32

Supplementary Table 2. Genotyping quality of multiplex PCR amplicons sequencing of replication cohort

**Items (N = 17870)**	**Mean ± Standard deviation**
Target average read length	148.82±0.39
Target average base quality	36.09±0.19
Target average insert size	213.08±4.85
Total reads mapping rate (%)	90.90±4.39
Target mean depth (×)	2598.61±1241.15
coverage ≥ 4 (%)	99.03±0.15
coverage ≥ 10 (%)	98.89±0.20
coverage ≥ 20 (%)	98.71±0.36
coverage ≥ 30 (%)	98.55±0.56

Supplementary Table 3. Exclusion criteria for quality control parameters for GWAS and replication cohorts

Exclusion criteria	GWAS cohort		Replication cohort	
	**Variants included**	**Sample included**	**Variants included**	**Sample included**
Original	80,993,588	4,496	90	17,870
MAF < 0.01*, non-autosnp	7,121,791	-	-	-
Multi-allelic variants	7,110,010			
Variants with high missing rate (> 5%)	-	-	90	-
Variants deviating from Hardy-Weinberg equilibrium in controls ($P < 1\times10^{-4}$)	6,910,086	-	90	-
Samples with high missing rate (> 5%)	-	4,496	-	17,633
Heterozygosity rate (deviating from mean $\pm$ 3 standard deviations)	-	4,480	-	-
Relatedness (identity by descent > 0.15)	-	4,450	-	-
Ancestry outliers	-	4,450	90	17,663
Final inclusion	6,910,086	4,450	90	17,663

*, GBA-L444P had MAF less than 0.01 in our GWAS and replication cohort but was still included due to its importance; MAF, minor allele frequency; autosnp, autosomal single nucleotide polymorphism.

Supplementary Table 4. Demographics of GWAS and replication cohorts

	GWAS cohort		Replication cohort	
	**Cases**	**Controls**	**Cases**	**Controls**
N	1,972	2,478	8,209	9,454
Age at recruitment	66.76 ± 7.08	62.32 ± 7.11	60.23 ± 11.20	64.29 ± 9.68
Age at onset	61.88 ± 6.93	-	57.77 ± 11.95	-
Sex (male/female)	991/981	1,010/1,468	4,489/3,720	4,480/4,974

Supplementary Table 5. Association results of tag SNPs within significant loci

Locus	Tag SNP	Position (hg19)	Effect allele	Other allele	GWAS cohort			Replication cohort			Combined		Info
					Beta	Se	*P*	Beta	Se	*P*	*P*	*P*het	
1	rs11557080	chr1:205737739	G	A	-0.249	0.046	6.08E-08	-0.206	0.024	1.80E-18	1.04E-24	0.3985	Known
2	rs12042698	chr1:225673483	G	A	-0.384	0.084	5.15E-06	-0.019	0.040	0.637	0.017	9.36E-05	New
3	rs1962462	chr3:116777735	T	C	-0.240	0.050	1.48E-06	0.011	0.025	0.657E	0.075	7.01E-06	New
4	rs76201116	chr3:117938522	C	T	0.656	0.146	7.18E-06	-0.026	0.070	0.710	0.109	2.57E-05	New
5	rs75364297	chr4:72209030	G	A	-0.576	0.120	1.64E-06	0.051	0.054	0.347	0.267	1.96E-06	New
6	rs356182	chr4:90626111	A	G	-0.344	0.049	1.52E-12	-0.342	0.025	2.56E-42	3.09E-53	0.970	Known
7	rs78246001	chr5:150753630	C	G	-0.343	0.075	4.20E-06	0.029	0.044	0.502	0.081	1.63E-05	New
8	rs997368	chr6:112243291	G	A	-0.160	0.046	5.37E-04	-0.109	0.023	3.39E-06	1.11E-08	0.326	Known
9	rs34594498	chr12:40646786	T	C	0.984	0.209	2.47E-06	0.855	0.108	2.64E-15	4.05E-20	0.586	Known
10	rs10147229	chr14:95384896	G	A	-0.289	0.065	7.99E-06	-0.026	0.033	0.433	0.007	2.83E-04	New
11	rs2251086	chr15:61997385	T	C	-0.191	0.061	1.77E-03	-0.158	0.030	1.56E-07	1.19E-09	0.622	Known
12	rs73615920	chr16:74114525	A	T	0.234	0.053	9.91E-06	-0.017	0.028	0.529	0.137	2.56E-05	New
13	rs11079147	chr17:53094754	G	A	0.200	0.045	7.33E-06	-0.007	0.023	0.742	0.085	3.29E-05	New
14	rs61204179	chr17:58139282	T	C	0.477	0.105	5.86E-06	0.242	0.054	7.91E-06	1.47E-09	0.047	New
15	rs34596409	chr19:13899223	A	G	-0.387	0.086	6.05E-06	-0.018	0.042	0.669	0.017	1.09E-04	New

Beta, regression coefficient; Se, standard error of beta; *P*het, the *P*-value of heterogeneity; Info, information about if the significant loci known.

Supplementary Table 6. Conditional analysis of loci reported to have multiple independent signals

Locus	Condition on	Most significant signal after condition				GWAS cohort					Replication cohort			*P*com	*P*het
		SNP	Position (hg19)	Effect allele	Other allele	R^2 with the tag SNP of the locus	Beta	Se	*P*	Considered an independent SNP	Beta	Se	*P*		
1	rs11557080	rs823127	1:205713139	A	G	0.51	1.306	0.071	1.68E-04	No	-	-	-	-	-
6	rs356182	rs4694012	4:90455143	A	G	<0.01	1.361	0.074	3.38E-05	No	-	-	-	-	-
9	rs34594498	rs33949390	12:40713845	A	G	<0.01	0.680	0.144	2.46E-06	Yes	0.506	0.076	3.09E-11	1.08E-15	0.343
9	rs34594498+ rs33949390	rs34778348	12:40757328	C	G	<0.01	0.493	0.110	7.36E-06	Yes	0.643	0.061	4.26E-26	3.22E-30	0.233
9	rs34594498+ rs33949390+ rs34778348	rs1491924	12:40587930	C	T	<0.01	0.437	0.108	5.06E-05	No	-	-	-	-	-

*P*com, the *P*-value of combined cohorts; *P*het, the *P*-value of heterogeneity.

Supplementary Table 7. Genome-wide gene-based association results of top 20 significant genes in GWAS cohort by MAGMA

Gene	Chr	Start	Stop	NSNPs	NPARAM	Zstat	*P*	Symbol
ENSG00000145335	4	90645250	90759466	237	16	6.107	5.07E-10	*SNCA*
ENSG00000117280	1	205737114	205744588	15	2	5.469	2.26E-08	*RAB7L1*
ENSG00000133065	1	205758221	205782876	28	4	5.341	4.63E-08	*SLC41A1*
ENSG00000069275	1	205681947	205719404	37	6	4.396	5.52E-06	*NUCKS1*
ENSG00000158715	1	205626979	205649587	44	9	4.090	2.16E-05	*SLC45A3*
ENSG00000166263	17	53046088	53241646	589	21	3.715	1.01E-04	*STXBP4*
ENSG00000268313	1	205682497	205684153	2	1	3.712	1.03E-04	*AC119673.1*
ENSG00000151892	10	117816444	118032979	569	51	3.696	1.10E-04	*GFRA1*
ENSG00000263563	17	21729601	21731762	6	2	3.650	1.31E-04	*UBBP4*
ENSG00000143184	1	168545711	168551315	24	6	3.623	1.45E-04	*XCL1*
ENSG00000186335	5	150694539	150727151	70	7	3.610	1.53E-04	*SLC36A2*
ENSG00000163067	2	95831177	95850065	16	5	3.598	1.60E-04	*ZNF2*
ENSG00000164023	4	108745719	108836203	151	24	3.573	1.76E-04	*SGMS2*
ENSG00000079308	2	218664512	218867718	533	70	3.570	1.78E-04	*TNS1*
ENSG00000144029	2	95752952	95815179	31	7	3.546	1.96E-04	*MRPS5*
ENSG00000175756	1	1309110	1310875	4	2	3.535	2.04E-04	*AURKAIP1*
ENSG00000242485	1	1337288	1342693	9	2	3.521	2.15E-04	*MRPL20*
ENSG00000155066	2	95940201	95957056	19	8	3.467	2.63E-04	*PROM2*
ENSG00000127419	4	926175	952444	60	15	3.435	2.96E-04	*TMEM175*
ENSG00000136867	9	115913222	115926417	61	11	3.429	3.02E-04	*SLC31A2*

Supplementary Table 8. Association results of reported GWAS SNPs in our GWAS cohort and replication cohorts and combined results

SNP ID	Position(hg19)	EA	OA	Nearest Gene	MAF_EAS	MAF_EUR	Effect_reported	GWAS cohort			Replication cohort			Combined			
								Beta	se	*P*	Beta	se	*P*	Beta	se	*P*	*P*het
rs356182	4:90626111	A	G	*SNCA*	0.332	0.650	-0.277	-0.344	0.049	1.52E-12	-0.342	0.025	2.56E-42	-0.343	0.022	3.09E-53	0.970
rs11557080	1:205737739	G	A	*RAB29*	0.457	0.855	-0.132	-0.249	0.046	6.08E-08	-0.206	0.024	1.80E-18	-0.215	0.021	1.04E-24	0.399
rs823118	1:205723572	C	T	*NUCKS1*	0.453	0.414	-0.107	-0.239	0.045	1.34E-07	-0.199	0.023	1.17E-17	-0.208	0.021	1.38E-23	0.429
rs823118*	1:205723572	C	T	*NUCKS1*	0.453	0.414	-0.107	-0.066	0.181	7.17E-01	-0.051	0.121	6.73E-01	-0.055	0.101	5.81E-01	0.946
rs10513789	3:182760073	T	G	*MCCC1*	0.421	0.797	0.149	0.111	0.045	1.29E-02	0.190	0.023	2.47E-16	0.173	0.021	3.51E-17	0.118
rs421016	1:155205043	G	A	*GBA*	0.001	0.001	-	1.458	0.513	4.50E-03	2.151	0.295	2.97E-13	1.979	0.256	9.82E-15	0.242
rs4698412	4:15737348	A	G	*BST1*	0.363	0.574	0.104	0.140	0.045	1.76E-03	0.153	0.023	2.16E-11	0.150	0.020	1.41E-13	0.802
rs2251086	15:61997385	T	C	*VPS13C*	0.162	0.149	-0.119	-0.191	0.061	1.77E-03	-0.158	0.030	1.56E-07	-0.164	0.027	1.19E-09	0.622
rs11158026	14:55348869	C	T	*GCH1*	0.433	0.609	0.084	0.106	0.044	1.60E-02	0.124	0.023	4.45E-08	0.120	0.020	2.33E-09	0.718
rs997368	6:112243291	G	A	*FYN*	0.341	0.128	-0.071	-0.160	0.046	5.37E-04	-0.109	0.023	3.39E-06	-0.119	0.021	1.11E-08	0.326
rs12456492	18:40673380	G	A	*RIT2*	0.376	0.366	0.098	0.118	0.044	7.74E-03	0.102	0.023	6.61E-06	0.106	0.020	1.78E-07	0.746
rs2248244	21:38852361	A	G	*DYRK1A*	0.350	0.341	0.071	0.092	0.045	3.87E-02	0.109	0.023	3.16E-06	0.105	0.021	3.60E-07	0.749
rs10847864	12:123326598	G	T	*HIP1R*	0.486	0.660	-0.148	-0.042	0.044	3.38E-01	-0.118	0.023	1.59E-07	-0.102	0.020	3.64E-07	0.119
rs34311866	4:951947	C	T	*TMEM175*	0.133	0.216	0.213	0.206	0.062	8.74E-04	0.115	0.032	3.61E-04	0.134	0.029	2.58E-06	0.192
rs10748818	10:104015279	G	A	*GBF1*	0.334	0.131	0.079	0.081	0.046	7.41E-02	0.103	0.024	1.37E-05	0.098	0.021	2.87E-06	0.673
rs6825004	4:77110365	G	C	*SCARB2*	0.383	0.248	-0.062	-0.038	0.045	4.01E-01	-0.113	0.024	1.47E-06	-0.097	0.021	3.13E-06	0.136
rs7938782	11:10558777	G	A	*RNF141*	0.151	0.100	-0.087	-0.098	0.061	1.08E-01	-0.130	0.031	3.42E-05	-0.123	0.028	9.96E-06	0.651
rs1474055	2:169110394	T	C	*STK39*	0.324	0.104	0.180	0.074	0.046	1.06E-01	0.094	0.024	6.98E-05	0.090	0.021	1.92E-05	0.698
rs1867598	5:60137959	G	A	*ELOVL7*	0.046	0.085	0.155	0.158	0.094	9.26E-02	0.175	0.045	1.07E-04	0.172	0.041	2.42E-05	0.873
rs4140646	6:27738801	A	G	*LOC100131289*	0.210	0.204	0.083	0.085	0.054	1.15E-01	0.106	0.028	1.88E-04	0.102	0.025	5.36E-05	0.731
rs10756907	9:17727065	G	A	*SH3GL2*	0.378	0.274	0.093	0.071	0.044	1.08E-01	0.083	0.023	3.41E-04	0.080	0.020	9.13E-05	0.808
rs12528068	6:72487762	T	C	*RIMS1*	0.074	0.252	0.066	0.177	0.076	1.97E-02	0.127	0.040	1.50E-03	0.138	0.035	9.67E-05	0.564
rs4653767	1:226916078	C	T	*ITPKB*	0.289	0.272	-0.083	-0.094	0.049	5.62E-02	-0.084	0.025	9.75E-04	-0.086	0.023	1.38E-04	0.848
rs199351	7:23300049	C	A	*GPNMB*	0.339	0.383	-0.102	-0.052	0.047	2.73E-01	-0.086	0.024	3.37E-04	-0.079	0.021	2.17E-04	0.519
rs1941685	18:31304318	G	T	*ASXL3*	0.113	0.435	-0.053	-0.163	0.075	3.05E-02	-0.109	0.038	3.64E-03	-0.120	0.034	3.65E-04	0.522
rs34025766	4:17968811	A	T	*LCORL*	0.141	0.091	-0.084	-0.137	0.064	3.31E-02	-0.091	0.034	6.66E-03	-0.101	0.030	6.97E-04	0.525
rs55818311	19:2341047	T	C	*SPPL2B*	0.315	0.693	-0.070	-0.071	0.046	1.22E-01	-0.068	0.024	4.28E-03	-0.069	0.021	1.16E-03	0.950
rs6500328	16:50736656	G	A	*NOD2*	0.185	0.413	-0.059	-0.113	0.057	4.53E-02	-0.075	0.029	9.18E-03	-0.083	0.026	1.24E-03	0.548
rs11150601	16:30977799	G	A	*SETD1A*	0.083	0.331	-0.091	-0.189	0.085	2.59E-02	-0.109	0.044	1.30E-02	-0.126	0.039	1.25E-03	0.402
rs2904880	16:28944396	C	G	*CD19*	0.084	0.316	-0.065	-0.059	0.079	4.61E-01	-0.130	0.041	1.39E-03	-0.115	0.036	1.47E-03	0.423

rs75859381	6:133210361	C	T	*RPS12*	0.035	0.039	0.221	-0.074	0.118	5.31E-01	0.230	0.060	1.09E-04	0.168	0.053	1.54E-03	0.021
rs11578699	1:171719769	T	C	*VAMP4*	0.176	0.161	-0.070	-0.041	0.062	5.13E-01	-0.089	0.030	2.99E-03	-0.080	0.027	3.03E-03	0.490
rs2042477	2:96000943	A	T	*KCNIP3*	0.442	0.234	-0.066	-0.106	0.044	1.60E-02	-0.047	0.023	3.66E-02	-0.060	0.020	3.09E-03	0.235
rs11610045	12:133063768	A	G	*FBRSL1*	0.133	0.491	0.060	0.096	0.067	1.54E-01	0.087	0.034	1.09E-02	0.089	0.030	3.54E-03	0.902
rs620513	8:16697593	T	G	*FGF20*	0.430	0.328	-0.086	-0.002	0.044	9.70E-01	-0.072	0.023	1.76E-03	-0.057	0.020	5.18E-03	0.161
rs10797576	1:232664611	T	C	*SIPA1L2*	0.142	0.100	0.111	0.011	0.060	8.57E-01	0.098	0.032	2.27E-03	0.079	0.028	5.41E-03	0.201
rs12147950	14:37989270	T	C	*MIPOL1*	0.490	0.393	-0.053	-0.097	0.043	2.58E-02	-0.045	0.023	4.84E-02	-0.056	0.020	5.44E-03	0.286
rs1450522	3:161077630	A	G	*SPTSSB*	0.464	0.689	-0.062	-0.073	0.044	1.00E-01	-0.050	0.023	2.92E-02	-0.054	0.020	7.05E-03	0.639
rs2269906	17:42294337	A	C	*UBTF*	0.222	0.713	0.063	-0.020	0.051	6.99E-01	0.079	0.027	3.20E-03	0.058	0.024	1.48E-02	0.088
rs3104783	16:52636242	C	A	*CASC16*	0.222	0.643	-0.067	-0.065	0.054	2.29E-01	-0.058	0.028	3.88E-02	-0.060	0.025	1.69E-02	0.912
rs12951632	17:40741013	C	T	*RETREG3*	0.430	0.238	-0.064	-0.022	0.044	6.14E-01	-0.054	0.023	1.68E-02	-0.047	0.020	1.87E-02	0.516
rs6808178	3:28705690	T	C	*LINC00693*	0.197	0.425	0.066	0.025	0.056	6.59E-01	0.068	0.029	1.77E-02	0.059	0.025	2.06E-02	0.490
rs62333164	4:170583157	A	G	*CLCN3*	0.072	0.304	-0.064	-0.041	0.088	6.43E-01	-0.106	0.046	2.12E-02	-0.092	0.041	2.41E-02	0.514
rs7134559	12:46419086	C	T	*SCAF11*	0.410	0.578	0.054	0.053	0.044	2.28E-01	0.043	0.023	6.16E-02	0.045	0.020	2.68E-02	0.836
rs3802920	11:133787001	T	G	*IGSF9B*	0.160	0.087	0.107	0.018	0.063	7.80E-01	0.075	0.032	1.98E-02	0.063	0.029	2.79E-02	0.417
rs6658353	1:161469054	C	G	*FCGR2A*	0.283	0.505	0.065	0.008	0.049	8.76E-01	0.059	0.025	1.88E-02	0.048	0.022	3.04E-02	0.348
rs61169879	17:59917366	C	T	*BRIP1*	0.447	0.731	-0.082	-0.039	0.044	3.71E-01	-0.043	0.023	6.05E-02	-0.042	0.020	3.77E-02	0.942
rs10221156	16:52969426	A	G	*CHD9*	0.037	0.094	-0.116	0.037	0.126	7.71E-01	-0.154	0.063	1.39E-02	-0.117	0.056	3.79E-02	0.177
rs11683001	2:102396963	A	T	*MAP4K4*	0.497	0.285	0.071	0.068	0.044	1.16E-01	0.033	0.022	1.38E-01	0.041	0.020	4.13E-02	0.474
rs9638616	7:70750493	T	G	*WBSCR17*	0.472	0.376	0.131	0.025	0.043	5.65E-01	0.045	0.023	5.13E-02	0.040	0.020	4.72E-02	0.681
rs11950533	5:134199105	A	C	*C5orf24*	0.263	0.194	-0.092	-0.019	0.049	7.05E-01	-0.050	0.025	4.57E-02	-0.043	0.022	5.05E-02	0.572
rs9568188	13:49927732	T	C	*CAB39L*	0.371	0.668	0.062	0.051	0.046	2.75E-01	0.036	0.023	1.15E-01	0.039	0.021	5.82E-02	0.777
rs55961674	3:122196892	T	C	*KPNA1*	0.122	0.096	0.086	-0.013	0.068	8.53E-01	0.073	0.034	3.30E-02	0.056	0.031	6.85E-02	0.260
rs12600861	17:7355621	C	A	*CHRNB1*	0.207	0.437	0.057	0.067	0.051	1.84E-01	0.034	0.026	1.92E-01	0.041	0.023	7.68E-02	0.560
rs11658976	17:44866805	A	G	*WNT3*	0.388	0.580	-0.062	0.054	0.044	2.20E-01	0.028	0.023	2.30E-01	0.033	0.021	1.04E-01	0.591
rs12283611	11:83487277	A	C	*DLG2*	0.235	0.462	-0.065	-0.078	0.050	1.17E-01	-0.024	0.026	3.45E-01	-0.035	0.023	1.21E-01	0.334
rs896435	10:15557406	T	C	*ITGA8*	0.428	0.584	0.074	-0.036	0.044	4.11E-01	0.048	0.023	3.35E-02	0.030	0.020	1.32E-01	0.087
rs13294100	9:17579690	G	T	*SH3GL2*	0.464	0.641	0.086	-0.006	0.044	8.94E-01	0.038	0.022	8.56E-02	0.029	0.020	1.43E-01	0.369
rs57891859	2:135464616	A	G	*TMEM163*	0.245	0.732	0.081	-0.014	0.051	7.89E-01	0.045	0.026	8.01E-02	0.033	0.023	1.47E-01	0.306
rs4771268	13:97865021	T	C	*MBNL2*	0.378	0.216	0.068	0.088	0.044	4.71E-02	0.010	0.023	6.49E-01	0.027	0.020	1.90E-01	0.119
rs6854006	4:77198054	T	C	*FAM47E-STBD1*	0.081	0.344	-0.091	0.128	0.076	9.12E-02	0.023	0.039	5.50E-01	0.045	0.035	1.93E-01	0.219
rs2086641	8:130901909	T	C	*FAM49B*	0.491	0.825	-0.061	-0.084	0.043	5.09E-02	-0.007	0.023	7.60E-01	-0.024	0.020	2.40E-01	0.112
rs979812	14:88464264	G	T	*GALC*	0.327	0.587	-0.061	0.036	0.046	4.33E-01	-0.037	0.024	1.23E-01	-0.021	0.021	3.15E-01	0.160
rs6476434	9:34046391	C	T	*UBAP2*	0.163	0.253	0.062	0.019	0.060	7.52E-01	0.029	0.031	3.50E-01	0.027	0.027	3.29E-01	0.888
rs850738	17:42434630	G	A	*FAM171A2*	0.470	0.443	0.071	-0.048	0.044	2.77E-01	0.035	0.022	1.17E-01	0.018	0.020	3.67E-01	0.092
rs2280104	8:22525980	T	C	*BIN3*	0.173	0.307	0.056	0.065	0.055	2.37E-01	0.011	0.029	6.91E-01	0.023	0.025	3.68E-01	0.387

rs12497850	3:48748989	G	T	*IP6K2*	0.032	0.339	-0.064	-0.053	0.111	6.33E-01	-0.041	0.054	4.50E-01	-0.043	0.049	3.74E-01	0.922
rs13117519	4:114369065	T	C	*CAMK2D*	0.090	0.110	0.088	0.031	0.079	6.94E-01	0.031	0.040	4.40E-01	0.031	0.035	3.86E-01	0.995
rs246814	5:75599208	T	C	*SV2C*	0.084	0.116	0.215	-0.144	0.074	5.18E-02	0.077	0.041	6.24E-02	0.025	0.036	4.94E-01	0.009
rs6497339	16:19277493	A	T	*SYT17*	0.014	0.402	0.063	-0.259	0.261	3.22E-01	0.141	0.129	2.71E-01	0.063	0.115	5.83E-01	0.169
rs4101061	4:77147969	A	G	*FAM47E*	0.224	0.685	-0.091	0.020	0.052	6.99E-01	-0.022	0.027	4.17E-01	-0.013	0.024	5.83E-01	0.476
rs76949143	7:66009851	A	T	*GS1-124K5.11*	0.155	0.154	-0.143	-0.102	0.065	1.17E-01	0.011	0.031	7.34E-01	-0.010	0.028	7.11E-01	0.118
rs3742785	14:75373034	C	A	*RPS6KL1*	0.219	0.280	-0.071	0.096	0.058	9.85E-02	-0.012	0.027	6.58E-01	0.008	0.025	7.61E-01	0.092
rs76904798	12:40614434	T	C	*LRRK2*	0.046	0.083	0.144	0.104	0.099	2.93E-01	-0.011	0.051	8.37E-01	0.014	0.046	7.64E-01	0.304
rs666463	17:76425480	T	A	*DNAH17*	0.039	0.108	-0.076	0.057	0.122	6.40E-01	-0.029	0.067	6.65E-01	-0.009	0.059	8.76E-01	0.537
rs11707416	3:151108965	A	T	*MED12L*	0.135	0.403	-0.063	0.112	0.061	6.50E-02	-0.035	0.032	2.69E-01	-0.003	0.028	9.04E-01	0.032
rs26431	5:102365794	G	C	*PAM*	0.447	0.325	-0.062	-0.109	0.045	1.46E-02	0.029	0.023	1.97E-01	0.001	0.020	9.60E-01	0.006
rs8087969	18:48683589	G	T	*MEX3C*	0.276	0.428	0.058	-0.059	0.048	2.23E-01	0.016	0.025	5.23E-01	0.0003	0.022	9.88E-01	0.169

*, conditioned on rs11557080 which was located within the same risk locus; EA, effect allele; OA, other allele; MAF, minor allele frequency; EUR, European; EAS, East Asian; Beta, regression coefficient; Se, standard error of beta; *P*het, the *P*-value of heterogeneity; MAF data was extracted from gnomAD database and reported beta was extracted from studies of European population (Nalls, 2019) and East Asian population (Foo, 2020).

Supplementary Table 9. Genetic correlation between Parkinson’s disease and other phenotypes in East Asian population

Trait	Genetic correlation, r_g	Se, r_g	Z, r_g	*P*, r_g	Observed H^2	Se, H^2	H^2, intercept	Se, H^2. intercept	Cross trait intercept	Se, Cross trait intercept	Source
Height	0.129	0.058	2.211	0.027	0.442	0.023	1.150	0.028	-0.0031	0.0061	BBJ
Type 2 Diabetes2	-0.142	0.067	-2.133	0.033	0.077	0.006	1.018	0.033	0.001	0.0051	BBJ-2020
Systemic lupus erythematosus	0.353	0.167	2.110	0.035	0.332	0.098	1.075	0.016	-0.0141	0.0064	GWAS catalog
Rheumatoid arthritis2	0.171	0.082	2.080	0.038	0.047	0.012	0.953	0.024	-0.0079	0.0045	GWAS catalog
G glutamyl transferase	0.192	0.093	2.076	0.038	0.113	0.028	1.032	0.027	-0.0075	0.0051	BBJ
Cheese consumption	0.306	0.159	1.929	0.054	0.022	0.003	0.998	0.008	0.0036	0.0051	GWAS catalog
Rheumatoid arthritis	0.262	0.138	1.900	0.057	0.021	0.008	0.994	0.014	-0.0038	0.0049	BBJ
Hematocrit	-0.209	0.114	-1.829	0.067	0.072	0.008	1.038	0.011	0.0114	0.0052	BBJ
Vegetable consumption	-0.350	0.192	-1.823	0.068	0.017	0.003	0.999	0.008	0.0044	0.005	GWAS catalog
Urolithiasis	-0.245	0.136	-1.805	0.071	0.018	0.003	1.001	0.009	0.0076	0.0045	BBJ
Hemoglobin	-0.200	0.119	-1.681	0.093	0.067	0.007	1.037	0.010	0.0104	0.0055	BBJ
Epithelial ovarian cancer	-0.559	0.337	-1.660	0.097	0.121	0.059	1.031	0.006	0.0177	0.0045	GWAS catalog
Red blood cell count	-0.150	0.096	-1.558	0.119	0.118	0.014	1.050	0.014	0.009	0.0055	BBJ
Graves’ disease	0.236	0.152	1.552	0.121	0.018	0.006	0.981	0.011	0.0015	0.0051	BBJ
Epilepsy	-0.602	0.405	-1.484	0.138	0.003	0.002	0.994	0.007	0.0038	0.0049	BBJ
Estimated glomerular filtration rate	-0.128	0.093	-1.388	0.165	0.099	0.009	1.092	0.013	0.0065	0.0058	BBJ
Yogurt consumption	0.178	0.129	1.382	0.167	0.032	0.004	0.999	0.008	-4.72E-05	0.0048	GWAS catalog
Peripheral artery disease	-0.216	0.157	-1.380	0.168	0.016	0.003	0.979	0.008	0.0028	0.0045	BBJ
Basophil count	0.188	0.140	1.347	0.178	0.068	0.017	1.079	0.011	-0.0017	0.005	BBJ
Breast cancer	-0.236	0.178	-1.328	0.184	0.030	0.006	0.977	0.008	0.0031	0.0052	BBJ
Tofu consumption	-0.229	0.173	-1.321	0.186	0.024	0.004	0.999	0.008	0.0094	0.0052	GWAS catalog
Aspartate aminotransferase	0.141	0.107	1.318	0.188	0.069	0.009	1.015	0.012	-0.0097	0.005	BBJ

Total bilirubin	0.166	0.129	1.287	0.198	0.059	0.019	1.038	0.017	-0.0067	0.0051	BBJ
Gut microbiome-Faecalibacterium	-0.370	0.288	-1.283	0.200	0.837	0.398	0.992	0.006	0.0088	0.0043	GWAS catalog
Gastric cancer	-0.212	0.169	-1.252	0.211	0.012	0.003	0.996	0.007	0.0046	0.0048	BBJ
Fish intake frequency	-0.164	0.133	-1.229	0.219	0.034	0.008	0.994	0.009	0.0104	0.0048	BBJ
smoking initiation	-0.124	0.101	-1.226	0.220	0.053	0.004	1.002	0.008	0.0034	0.0053	BBJ
Monocyte count	-0.147	0.123	-1.195	0.232	0.088	0.013	1.056	0.011	0.0021	0.0049	BBJ
Type 2 Diabetes1	-0.088	0.078	-1.142	0.254	0.105	0.009	1.043	0.028	0.0015	0.0054	BBJ-2019
Serum creatinine	0.100	0.089	1.115	0.265	0.103	0.009	1.078	0.014	-0.0061	0.0058	BBJ
Natto consumption	-0.144	0.129	-1.112	0.266	0.032	0.005	1.003	0.008	0.0039	0.0045	GWAS catalog
Smoking cessation	0.173	0.161	1.071	0.284	0.035	0.007	1.001	0.007	0.0008	0.0045	BBJ
Disc area	-0.305	0.288	-1.061	0.289	0.150	0.074	1.003	0.007	-0.0013	0.0045	GWAS catalog
Chronic obstructive pulmonary disease	-0.155	0.148	-1.050	0.294	0.012	0.003	0.987	0.008	0.0025	0.0046	BBJ
Glaucoma	0.154	0.148	1.046	0.296	0.014	0.002	1.001	0.007	-0.0063	0.0047	BBJ-2020
Chronic hepatitis B	-0.222	0.213	-1.041	0.298	0.008	0.004	0.985	0.008	-0.0015	0.0049	BBJ
Glaucoma2	0.128	0.125	1.026	0.305	0.114	0.013	0.986	0.007	-0.0042	0.0047	GWAS catalog
Alanine aminotransferase	0.122	0.120	1.023	0.306	0.063	0.009	1.014	0.010	-0.0023	0.005	BBJ
Interventricular septum thickness	0.256	0.252	1.016	0.310	0.054	0.025	1.022	0.007	-0.0073	0.005	BBJ
Drug eruption	0.500	0.495	1.010	0.312	0.002	0.002	0.985	0.008	-0.004	0.0049	BBJ
Hemoglobin concentration	-0.099	0.101	-0.988	0.323	0.067	0.006	1.065	0.010	0.0059	0.0046	GWAS catalog
Platelet count	-0.078	0.080	-0.973	0.331	0.158	0.026	1.054	0.023	0.0011	0.0053	BBJ
Cigarettes per day	0.140	0.146	0.958	0.338	0.071	0.011	1.012	0.014	-0.0028	0.0051	BBJ
Blood urea nitrogen	-0.098	0.102	-0.956	0.339	0.074	0.008	1.061	0.010	0.0047	0.0051	BBJ
Non-albumin protein	-0.107	0.114	-0.935	0.350	0.095	0.011	1.089	0.026	0.0038	0.0056	BBJ
Gut microbiome-unassigned genus belonging to family Clostridiales	0.262	0.289	0.907	0.364	0.775	0.426	0.994	0.007	-0.0008	0.0046	GWAS catalog
Mean arterial pressure	0.087	0.096	0.907	0.365	0.073	0.008	1.050	0.010	-0.0088	0.0051	BBJ
Neutrophil count	-0.103	0.116	-0.884	0.377	0.117	0.015	1.013	0.009	0.0029	0.0052	BBJ

Creatine kinase	-0.086	0.098	-0.880	0.379	0.095	0.011	1.056	0.039	0.0022	0.0045	BBJ
Relative wall thickness	0.175	0.201	0.867	0.386	0.083	0.027	1.010	0.008	-0.0002	0.0045	BBJ
Lung cancer	0.234	0.276	0.849	0.396	0.005	0.002	0.998	0.007	-0.0038	0.0046	BBJ
Albumin/globulin ratio	0.091	0.108	0.847	0.397	0.092	0.011	1.067	0.021	-0.0032	0.0051	BBJ
Gut microbiome-Sutterella	0.469	0.573	0.818	0.413	0.263	0.415	0.988	0.007	-0.0047	0.0041	GWAS catalog
Diastolic blood pressure	0.082	0.104	0.788	0.431	0.060	0.006	1.041	0.009	-0.0086	0.0051	BBJ
Cataract	0.166	0.215	0.773	0.439	0.007	0.002	1.052	0.008	0.0007	0.0054	BBJ
Systolic blood pressure	0.072	0.094	0.770	0.442	0.077	0.008	1.051	0.011	-0.0071	0.0052	BBJ
Menopause	-0.095	0.126	-0.751	0.453	0.115	0.018	0.990	0.008	0.008	0.0045	BBJ
Coronary artery disease	0.056	0.078	0.720	0.472	0.072	0.009	1.000	0.013	-0.0021	0.005	BBJ
Alkaline phosphatase	-0.074	0.104	-0.712	0.477	0.090	0.024	1.105	0.033	-0.0002	0.0052	BBJ
Lactate dehydrogenase	0.108	0.154	0.698	0.485	0.027	0.009	1.105	0.042	0.0028	0.0052	BBJ
Triglyceride	-0.070	0.100	-0.698	0.485	0.123	0.033	1.018	0.018	0.004	0.0053	BBJ
Cup area	0.166	0.262	0.634	0.526	0.115	0.058	1.000	0.007	0.0023	0.0043	GWAS catalog
Lymphocyte count	-0.079	0.126	-0.630	0.529	0.086	0.011	1.028	0.009	0.0062	0.0053	BBJ
White blood cell count	-0.056	0.092	-0.609	0.543	0.104	0.010	1.036	0.011	0.0042	0.0052	BBJ
Open-angle glaucoma	0.093	0.158	0.586	0.558	0.125	0.023	1.103	0.008	-0.004	0.0049	BBJ-2018
Left ventricular mass	0.095	0.162	0.585	0.559	0.153	0.027	1.000	0.007	-0.0079	0.0047	BBJ
Left ventricular mass index	0.104	0.182	0.574	0.566	0.118	0.027	1.000	0.007	-0.0084	0.0045	BBJ
Arrhythmia	0.062	0.109	0.567	0.571	0.029	0.005	1.013	0.014	-0.0028	0.0047	BBJ
Drinks per week	0.070	0.124	0.563	0.574	0.087	0.055	0.982	0.015	-0.0027	0.005	GWAS catalog
Age of smoking initiation	0.122	0.225	0.545	0.586	0.044	0.016	0.998	0.007	0.0013	0.0049	BBJ
Potassium	0.059	0.112	0.522	0.601	0.054	0.007	1.018	0.011	-0.0003	0.0048	BBJ
Mosaic loss of chromosome Y	0.059	0.116	0.513	0.608	0.076	0.013	1.018	0.013	-0.0027	0.0048	BBJ
Green tea consumption	0.089	0.175	0.511	0.610	0.019	0.004	1.001	0.008	1.36E-05	0.0054	GWAS catalog
Osteoporosis	0.107	0.210	0.507	0.612	0.008	0.002	1.001	0.007	-0.0073	0.005	BBJ

Gut microbiome-Bifidobacterium	0.737	1.454	0.507	0.612	0.158	0.419	0.993	0.007	-0.0042	0.004	GWAS catalog
Eosinophil count	-0.059	0.117	-0.504	0.615	0.110	0.015	1.032	0.011	0.0047	0.005	BBJ
Endometrial cancer	-0.133	0.267	-0.496	0.620	0.010	0.005	0.968	0.007	-0.0009	0.0045	BBJ
Membranous nephropathy	0.784	1.591	0.493	0.622	0.148	0.224	1.028	0.008	-0.0161	0.0051	GWAS catalog
Body mass index	-0.037	0.077	-0.477	0.633	0.170	0.008	1.066	0.014	-0.0079	0.0055	BBJ
Small whole fish consumption	0.070	0.151	0.461	0.645	0.029	0.004	1.000	0.008	-0.0005	0.0049	GWAS catalog
facial pigmentation measurement UV light	0.085	0.184	0.460	0.645	0.216	0.050	1.006	0.008	-0.0026	0.0053	GWAS catalog
Activated partial thromboplastin time	-0.089	0.197	-0.449	0.653	0.046	0.015	1.035	0.014	-0.0044	0.0051	BBJ
Sodium	0.054	0.122	0.445	0.656	0.055	0.007	1.014	0.010	-0.0023	0.0052	BBJ
Hemoglobin A1c	-0.058	0.131	-0.442	0.659	0.105	0.019	1.041	0.011	-0.0019	0.0046	BBJ
Colorectal cancer	-0.077	0.174	-0.439	0.661	0.080	0.016	1.211	0.008	0.0022	0.0049	BBJ
Phosphorus	0.081	0.187	0.434	0.665	0.048	0.012	1.024	0.009	0.0005	0.0046	BBJ
Total cholesterol	-0.048	0.111	-0.433	0.665	0.051	0.012	1.069	0.028	0.0061	0.005	BBJ
Albumin	0.053	0.124	0.432	0.666	0.060	0.007	1.011	0.010	-0.0015	0.005	BBJ
Prothrombin time	0.075	0.176	0.423	0.672	0.056	0.024	1.040	0.032	-0.0096	0.0052	BBJ
Posterior wall thickness	0.069	0.173	0.396	0.692	0.104	0.024	1.003	0.007	-0.0019	0.0045	BBJ
Cirrhosis	0.139	0.354	0.394	0.694	0.003	0.003	1.007	0.008	0.0012	0.0049	BBJ
Congestive heart failure	-0.072	0.186	-0.390	0.697	0.011	0.002	1.014	0.007	-0.0014	0.005	BBJ
Chloride	0.045	0.116	0.387	0.699	0.058	0.007	1.059	0.011	0.0005	0.0048	BBJ
Fractional shortening	-0.093	0.243	-0.384	0.701	0.075	0.024	1.006	0.008	0.0061	0.0049	BBJ
Menarche	-0.039	0.105	-0.371	0.711	0.110	0.010	1.021	0.007	0.0005	0.0051	BBJ
Cervical cancer	-0.092	0.248	-0.370	0.711	0.015	0.006	0.986	0.008	-0.003	0.0049	BBJ
Left ventricular internal dimension in diastole	-0.062	0.172	-0.361	0.718	0.140	0.024	0.998	0.008	-0.001	0.0045	BBJ
Gut microbiome-Dorea	-0.172	0.493	-0.349	0.728	0.293	0.421	0.992	0.007	-0.0066	0.0048	GWAS catalog
Uric acid	0.027	0.079	0.348	0.728	0.159	0.052	0.996	0.024	-0.0074	0.0054	BBJ
Nephrotic syndrome	-0.112	0.322	-0.347	0.728	0.004	0.002	0.992	0.008	0.0011	0.0049	BBJ

Gut microbiome-Ruminococcus belonging to family Lachnospiraceae	-0.188	0.545	-0.345	0.730	0.241	0.427	1.008	0.007	0.0074	0.0044	GWAS catalog
Pulmonary tuberculosis	-0.113	0.336	-0.337	0.736	0.003	0.002	0.992	0.007	-0.0023	0.0048	BBJ
Coffee consumption	0.035	0.105	0.330	0.741	0.063	0.016	0.980	0.013	0.0025	0.0048	GWAS catalog
Keloid	0.067	0.212	0.317	0.751	0.007	0.002	0.979	0.007	-0.0013	0.0051	BBJ
Pulse pressure	0.034	0.110	0.307	0.759	0.053	0.005	1.035	0.009	-0.0004	0.0052	BBJ
Pollinosis	-0.063	0.217	-0.291	0.771	0.007	0.002	1.000	0.008	0.0023	0.0048	BBJ
Total protein	-0.029	0.120	-0.242	0.809	0.078	0.008	1.069	0.018	-0.0001	0.0056	BBJ
Hematological malignancy	-0.226	0.983	-0.230	0.818	0.001	0.002	0.986	0.007	0.0067	0.005	BBJ
Atrial Fibrillation	0.029	0.128	0.223	0.824	0.132	0.025	1.218	0.013	-0.0046	0.0053	BBJ
Facia pigmentation polar light	-0.039	0.180	-0.215	0.830	0.143	0.030	1.003	0.007	-0.003	0.0049	GWAS catalog
Esophageal cancer	-0.033	0.163	-0.205	0.838	0.013	0.005	0.957	0.009	0.0004	0.0046	BBJ
Mean corpuscular hemoglobin	-0.016	0.079	-0.201	0.841	0.158	0.025	1.045	0.021	0.0041	0.0056	BBJ
C-reactive protein	-0.048	0.242	-0.200	0.842	0.019	0.009	1.177	0.012	-0.0055	0.0049	BBJ
Atopic dermatitis	0.029	0.154	0.190	0.849	0.012	0.003	0.975	0.008	0.0015	0.0048	BBJ
Gut microbiome-Parabacteroides	-0.053	0.285	-0.186	0.853	0.735	0.418	0.982	0.007	-0.0002	0.0048	GWAS catalog
Milk consumption	0.021	0.116	0.181	0.857	0.036	0.004	0.998	0.008	0.0061	0.0054	GWAS catalog
High-density-lipoprotein cholesterol	0.020	0.110	0.180	0.857	0.107	0.038	1.120	0.060	-0.0019	0.0053	BBJ
Blood sugar	-0.021	0.122	-0.175	0.861	0.050	0.009	1.019	0.010	-6.36E-05	0.0048	BBJ
Sweet taste preference	0.029	0.178	0.166	0.869	0.231	0.106	0.987	0.012	-0.0028	0.0062	GWAS catalog
Calcium	-0.026	0.162	-0.160	0.873	0.050	0.010	1.011	0.009	-0.0011	0.0052	BBJ
Ejection fraction	-0.037	0.236	-0.156	0.876	0.079	0.026	1.002	0.008	0.004	0.0048	BBJ
Mean corpuscular hemoglobin concentration	-0.020	0.134	-0.148	0.883	0.053	0.010	1.055	0.014	0.0009	0.0058	BBJ
Meat consumption	-0.022	0.153	-0.145	0.885	0.023	0.004	1.001	0.008	-2.52E-05	0.0047	GWAS catalog
Chronic hepatitis C	-0.029	0.207	-0.142	0.887	0.008	0.003	0.996	0.007	0.0039	0.005	BBJ
Cerebral aneurysm	0.023	0.167	0.137	0.891	0.015	0.003	0.988	0.008	-0.0068	0.0047	BBJ

Ovarian cancer	-0.045	0.462	-0.097	0.923	0.003	0.004	0.970	0.007	-0.007	0.0048	BBJ
Central corneal thickness	0.016	0.167	0.096	0.923	0.272	0.060	1.000	0.008	0.003	0.0047	GWAS catalog
hepatocellular carcinoma	-0.027	0.309	-0.089	0.929	0.004	0.003	0.992	0.007	-0.0048	0.0043	BBJ
Fibrinogen	-0.031	0.353	-0.086	0.931	0.026	0.024	1.006	0.007	0.0003	0.0048	BBJ
Prostate cancer	-0.008	0.127	-0.065	0.948	0.045	0.007	0.978	0.021	-0.0035	0.0048	BBJ
Left ventricular internal dimension in systole	0.011	0.174	0.063	0.950	0.144	0.026	0.998	0.007	-0.005	0.0046	BBJ
Mean corpuscular volume	0.005	0.077	0.058	0.954	0.178	0.026	1.053	0.021	0.0032	0.0054	BBJ
Low-density-lipoprotein cholesterol	0.005	0.148	0.037	0.971	0.055	0.021	1.056	0.034	-0.0004	0.0049	BBJ
Ischemic stroke	0.004	0.142	0.030	0.976	0.016	0.003	1.021	0.008	-0.0017	0.0049	BBJ
Gut microbiome-unassigned genus belonging to family Lachnospiraceae	-0.008	0.340	-0.023	0.982	0.563	0.430	0.996	0.008	0.0007	0.0048	GWAS catalog
Zinc sulfate turbidity test	0.003	0.255	0.010	0.992	0.082	0.041	1.023	0.009	-0.0023	0.0048	BBJ
Asthma	-0.001	0.142	-0.004	0.997	0.023	0.003	1.005	0.009	0.0001	0.0052	BBJ
Biliary tract cancer	NA	NA	NA	NA	-0.004	0.002	1.003	0.007	0.0007	0.0049	BBJ
E/A ratio	NA	NA	NA	NA	NA	NA	NA	NA	NA	NA	BBJ
Endometriosis	NA	NA	NA	NA	-0.002	0.004	0.986	0.007	0.0013	0.0045	BBJ
Gut microbiome-Bacteroides	NA	NA	NA	NA	-0.459	0.399	1.006	0.006	-0.006	0.0047	GWAS catalog
Gut microbiome-Blautia	NA	NA	NA	NA	-0.302	0.407	1.002	0.007	-0.0041	0.0049	GWAS catalog
Gut microbiome-Coprococcus	NA	NA	NA	NA	NA	NA	NA	NA	NA	NA	GWAS catalog
Gut microbiome-Eubacterium belonging to family Erysipelotrichaceae	NA	NA	NA	NA	-0.225	0.391	1.002	0.007	-0.0102	0.0043	GWAS catalog
Gut microbiome-Oscillospira	NA	NA	NA	NA	-0.567	0.398	1.013	0.007	0.0026	0.0047	GWAS catalog
Gut microbiome-Prevotella	NA	NA	NA	NA	-0.607	0.382	1.004	0.007	0.0031	0.0045	GWAS catalog
Gut microbiome-Ruminococcus belonging to family Erysipelotrichaceae	NA	NA	NA	NA	NA	NA	NA	NA	NA	NA	GWAS catalog
Gut microbiome-Streptococcus	NA	NA	NA	NA	-0.734	0.412	1.011	0.006	-0.0012	0.0044	GWAS catalog
Gut microbiome-unclassified genus belonging to family Clostridiaceae	NA	NA	NA	NA	-0.715	0.404	1.008	0.007	0.005	0.0046	GWAS catalog

Gut microbiome-unclassified genus belonging to family Erysipelotrichaceae	NA	NA	NA	NA	NA	NA	NA	NA	NA	NA	GWAS catalog
Gut microbiome-unclassified genus belonging to family Lachnospiraceae	NA	NA	NA	NA	-0.100	0.397	1.008	0.007	0.0002	0.0051	GWAS catalog
Gut microbiome-unclassified genus belonging to family Ruminococcaceae	NA	NA	NA	NA	NA	NA	NA	NA	NA	NA	GWAS catalog
Gut microbiome-unclassified genus belonging to the order Clostridiales	NA	NA	NA	NA	NA	NA	NA	NA	NA	NA	GWAS catalog
Interstitial lung disease	NA	NA	NA	NA	-0.001	0.002	1.007	0.008	-0.0053	0.0046	BBJ
Pancreatic cancer	NA	NA	NA	NA	NA	NA	NA	NA	NA	NA	BBJ
Periodontal disease	NA	NA	NA	NA	-0.001	0.002	1.019	0.007	-0.0013	0.0045	BBJ
Tuberculosis	NA	NA	NA	NA	-0.048	0.208	1.031	0.007	0.0064	0.0053	GWAS catalog
Tuberculosis2	NA	NA	NA	NA	NA	NA	NA	NA	NA	NA	BBJ

BBJ, BioBank Japan Project.

Supplementary Table 10. Mendelian randomization study of Parkinson's disease and genetic correlated traits

Exposure	Outcome	NSNPs	MR Egger			Inverse variance weighted		
			**Beta**	**Se**	***P***	**Beta**	**Se**	***P***
Parkinson's disease	Gamma-glutamyl transferase	7	-0.081	0.046	0.137	-0.024	0.013	0.065
Gamma-glutamyl transferase	Parkinson's disease	22	-0.047	0.031	0.150	-0.009	0.012	0.430
Parkinson's disease	Height	10	0.0003	0.024	0.989	0.005	0.008	0.526
Heigh	Parkinson's disease	25	0.004	0.017	0.815	0.004	0.007	0.547
Parkinson's disease	Type 2 diabetes	10	0.016	0.030	0.620	0.004	0.016	0.815
Type 2 diabetes	Parkinson's disease	25	-0.017	0.032	0.587	0.006	0.015	0.682
Parkinson's disease	Systemic lupus erythematosus	7	-0.121	0.356	0.748	-0.121	0.356	0.748
Systemic lupus erythematosus	Parkinson's disease	20	0.010	0.217	0.965	0.020	0.075	0.793
Parkinson's disease	Rheumatoid arthritis	11	-0.012	0.051	0.813	-0.001	0.027	0.983
Rheumatoid arthritis	Parkinson's disease	26	-0.004	0.049	0.939	-0.009	0.027	0.745

NSNPs, number of included SNPs; MR, Mendelian randomization; Beta, regression coefficient; Se, standard error of beta.

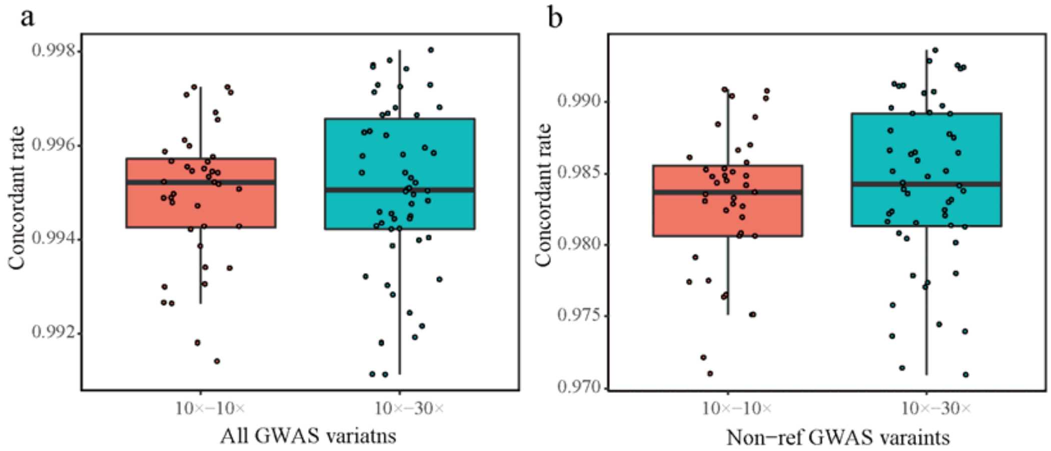

Supplementary Figure 1. Comparison of the genotype concordance between matched samples applied WGS with a medium degree of depth setting (10× depth) and a high degree of depth setting (30× depth). The box center represents the group median; the box edges reflect the value of the first and third quartile cutoffs, respectively; the length of the box is the interquartile range; the whiskers indicate the value for outliers (two interquartile ranges away from the median).

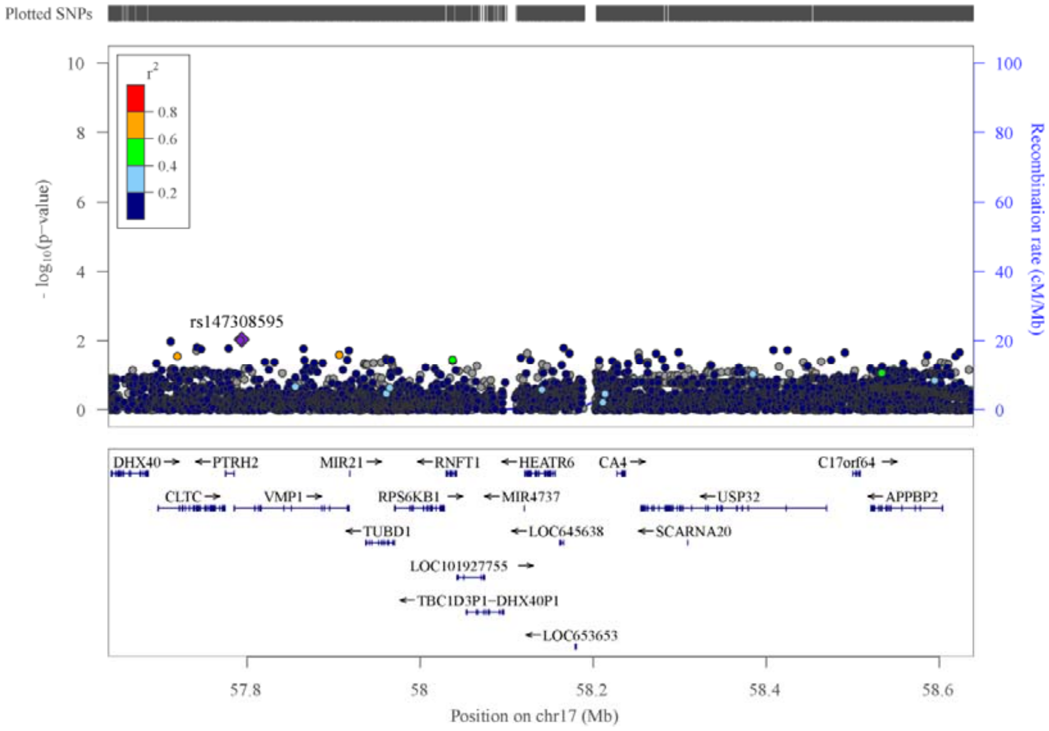

Supplementary Figure 2. Regional plot of the HEATR6 locus in the European-ancestry PD results. Reference data of European-ancestry population from 1000 Genomes were used for linkage disequilibrium calculation.

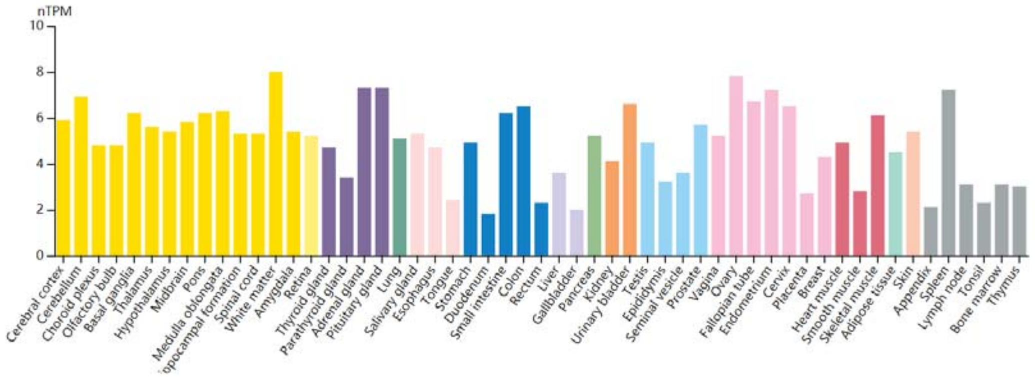

Supplementary Figure 3. Consensus RNA expression data of *HEATR6* from the Human Protein Atlas. The consensus dataset consists of normalized expression levels for 55 tissue types, created by combining the HPA and GTEx transcriptomics datasets using the internal normalization pipeline. Color-coding is based on different tissues, each consisting of tissues with functional features in common.

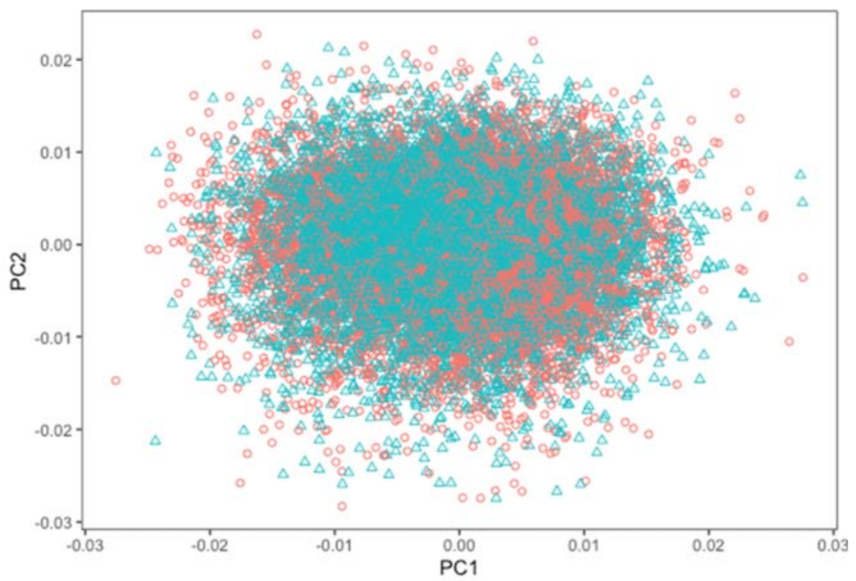

Supplementary Figure 4. PCA of our 8,209 PD cases and 9,454 controls in replication cohort using 37 non-associated SNPs.
