# Supplementary material for: Genome-wide association study using whole-genome sequencing identifies risk loci for Parkinson’s disease in Chinese population
Source: NPJ Parkinsons Dis. 2023 Feb 9;9:22. doi: 10.1038/s41531-023-00456-6 (PMC9911746; doi:10.1038/s41531-023-00456-6)
Supplement: Supplementary file 2 — Reporting Summary [file 41531_2023_456_MOESM2_ESM.pdf]

nature portfolio

Corresponding author(s): Beisha Tang

Last updated by author(s): Dec 20, 2022

Reporting Summary

Nature Portfolio wishes to improve the reproducibility of the work that we publish. This form provides structure for consistency and transparency in reporting. For further information on Nature Portfolio policies, see our Editorial Policies and the Editorial Policy Checklist.

Statistics

For all statistical analyses, confirm that the following items are present in the figure legend, table legend, main text, or Methods section.

n/a	Confirmed	
☐	☒	The exact sample size (*n*) for each experimental group/condition, given as a discrete number and unit of measurement
☐	☒	A statement on whether measurements were taken from distinct samples or whether the same sample was measured repeatedly
☐	☒	The statistical test(s) used AND whether they are one- or two-sided<br>*Only common tests should be described solely by name; describe more complex techniques in the Methods section.*
☐	☒	A description of all covariates tested
☐	☒	A description of any assumptions or corrections, such as tests of normality and adjustment for multiple comparisons
☐	☒	A full description of the statistical parameters including central tendency (e.g. means) or other basic estimates (e.g. regression coefficient) AND variation (e.g. standard deviation) or associated estimates of uncertainty (e.g. confidence intervals)
☐	☒	For null hypothesis testing, the test statistic (e.g. *F*, *t*, *r*) with confidence intervals, effect sizes, degrees of freedom and *P* value noted<br>*Give P values as exact values whenever suitable.*
☒	☐	For Bayesian analysis, information on the choice of priors and Markov chain Monte Carlo settings
☒	☐	For hierarchical and complex designs, identification of the appropriate level for tests and full reporting of outcomes
☐	☒	Estimates of effect sizes (e.g. Cohen's *d*, Pearson's *r*), indicating how they were calculated

Our web collection on statistics for biologists contains articles on many of the points above.

Software and code

Policy information about availability of computer code

Data collection	No software was used.
Data analysis	1. Variant calling was conducted using GATK4 version 4.1.4.0.<br>2. Clean reads were aligned to the GRCh37 human reference genome (hg19) using the BWA mem tool version 0.7.17.<br>3. SAMtools version 1.7 was used convert SAM files into BAM files.<br>4. LD-based genotype refinement for low-confidence genotypes and missing sites in WGS data using BEAGLE v5.1.<br>5. For individual and variant quality control of WGS data, as well as logistic regression analysis in GWAS, PLINK v1.9 was used.<br>6. Meta-analysis to combine the association results from the GWAS and replication study was conducted using METAL.<br>7. MAGMA version 1.07b was used for gene-based analysis of common variants.<br>8. Linkage disequilibrium score regression (LDSC) version 1.0.1 and univariate genome-wide complex trait analysis- genomic-relatedness-based restricted maximum-likelihood (GCTA-GREML) version 1.92.2beta with default parameters was used to estimate the heritability of PD.<br>9. POPCORN version 0.9.6 was used to investigate the genetic correlation of PD between populations.<br>10. Mendelian randomisation was performed using the MendelianRandomization package in R.<br>11. Rolygenic risk score analysis was performed with PRSice2.

For manuscripts utilizing custom algorithms or software that are central to the research but not yet described in published literature, software must be made available to editors and reviewers. We strongly encourage code deposition in a community repository (e.g. GitHub). See the Nature Portfolio guidelines for submitting code & software for further information.

Data

Policy information about availability of data

All manuscripts must include a data availability statement. This statement should provide the following information, where applicable:

- Accession codes, unique identifiers, or web links for publicly available datasets
- A description of any restrictions on data availability
- For clinical datasets or third party data, please ensure that the statement adheres to our policy

DATA AVAILABILITY
According to national legislation/guidelines, specifically theAdministrative Regulations of the People's Republic of China on HumanGenetic Resources (http://www.gov.cn/zhengce/content/2019-06/10/content_5398829.htm, http://english.www.gov.cn/policies/latest_releases/2019/06/10/content_281476708945462.htm), no additional raw data is available at this time. Data of this project can be accessed after an approval application to the China National Genebank(CNGB, https://db.cngb.org/cnsa/). Please refer to https://db.cngb.org/, oremail: gro.bgnc@bdBGNC for detailed application guidance. The accession code CNP0003805 should be included in the application.

Human research participants

Policy information about studies involving human research participants and Sex and Gender in Research.

Reporting on sex and gender	Sex was used to reflect biological assignment at birth as patient reported or documented in medical records.
Population characteristics	The final subjects included in the statistical analysis after quality control encompassed 1,972 cases (the mean age at recruitment, 66.76±7.08 years; the mean age at onset, 61.88 ± 6.93 years) and 2,478 controls (the mean age at recruitment, 62.32 ± 7.11 years) in the GWAS cohort, and 8,209 cases (the mean age at recruitment, 60.23 ± 11.20 years; the mean age at onset, 57.77 ± 11.95 years) and 9,454 controls (the mean age at recruitment, 64.29 ± 9.68 years) in the replication cohort
Recruitment	Patients with PD and control subjects free from neurological disorders were recruited from study groups participating in the Parkinson's Disease and Movement Disorders Multicenter Database and Collaborative Network in China (http://pd-mdcnc.com) and centres across China.
Ethics oversight	The study was approved by the ethics committees of Xiangya Hospital of Central South University and other study groups in PD-MDCNC: Xuanwu Hospital of Capital Medical University; Ruijin Hospital of Shanghai Jiao Tong University School of Medicine; Affiliated Brain Hospital of Nanjing Medical University; Union Hospital of Huazhong University of Science and Technology; the First Affiliated Hospital of Zhengzhou University and First Affiliated Hospital of Sun Yat-sen University.

Note that full information on the approval of the study protocol must also be provided in the manuscript.

Field-specific reporting

Please select the one below that is the best fit for your research. If you are not sure, read the appropriate sections before making your selection.

☒ Life sciences ☐ Behavioural & social sciences ☐ Ecological, evolutionary & environmental sciences

For a reference copy of the document with all sections, see nature.com/documents/nr-reporting-summary-flat.pdf

Life sciences study design

All studies must disclose on these points even when the disclosure is negative.

Sample size	The total sample size of this study exceeded 20,000 which had sufficient power to catch genome-wide significant signals according to previous experiences, however, we only performed whole genome sequencing with around 2,000 cases and 2,500 healthy controls. Under the current sample size in GWAS, the statistical power could reach 70% when the MAFs of the target variants were 0.05 and the odd ratios were 1.35.
Data exclusions	Variant quality control was accomplished by keeping all autosomal SNPs with minor allele frequency ≥ 0.01 which may have sufficient power to detect the associations given the sample size in GWAS cohort,13 while removing variants deviating from the Hardy-Weinberg equilibrium (P < 1×10−4). Individuals and variants that passed the quality control thresholds were subjected to further analyses. Individuals were excluded if they showed conflicting sex assignments between inferred sex and self-reported sex, deviating heterozygosity/genotype calls (±3 standard deviations), or cryptic relatedness (identity by descent > 0.15). The remaining samples were assessed for population outliers and stratification by principal component analysis using PLINK v1.9, and those that showed divergent ancestry were excluded. For the replication cohort, variants with low-quality genotypes (Phred-scaled genotype quality score 30), low call rates (missing rate > 5%), or departure from Hardy-Weinberg equilibrium (P < 1×10−4) were excluded. Samples with low genotype call rates (missing rate > 5%) were removed from further analysis. We also did principal component analysis using genotyped variants not associating with PD and excluded the outliers
Replication	A total of 8,209 cases and 9,454 controls formed the replication cohort to validate the finding in GWAS and the reported GWAS signals in European-ancestry population.

Randomization	This is a case-control study, and the subjects were randomly recruited from study groups participating in the Parkinson's Disease and Movement Disorders Multicenter Database and Collaborative Network in China (http://pd-mdcnc.com) and centres across China.
Blinding	The investigators were blinded to genotypes when carrying out any experiments.

Reporting for specific materials, systems and methods

We require information from authors about some types of materials, experimental systems and methods used in many studies. Here, indicate whether each material, system or method listed is relevant to your study. If you are not sure if a list item applies to your research, read the appropriate section before selecting a response.

Materials & experimental systems

n/a	Involved in the study
☒	☐ Antibodies
☒	☐ Eukaryotic cell lines
☒	☐ Palaeontology and archaeology
☒	☐ Animals and other organisms
☐	☒ Clinical data
☒	☐ Dual use research of concern

Methods

n/a	Involved in the study
☒	☐ ChIP-seq
☒	☐ Flow cytometry
☒	☐ MRI-based neuroimaging

Clinical data

Policy information about clinical studies

All manuscripts should comply with the ICMJE guidelines for publication of clinical research and a completed CONSORT checklist must be included with all submissions.

Clinical trial registration	n/a
Study protocol	n/a
Data collection	n/a
Outcomes	n/a
